# Correction: Filippou et al. Transcriptomic Analysis Reveals Molecular Mechanisms Underpinning Mycovirus-Mediated Hypervirulence in *Beauveria bassiana* Infecting *Tenebrio molitor*. *J. Fungi* 2025, *11*, 63

**DOI:** 10.3390/jof11110782

**Published:** 2025-10-30

**Authors:** Charalampos Filippou, Robert H. A. Coutts, Ioly Kotta-Loizou, Sam El-Kamand, Alexie Papanicolaou

**Affiliations:** 1Department of Medicine, School of Medicine, European University Cyprus, 2404 Nicosia, Cyprus; 2Department of Clinical, Pharmaceutical and Biological Science, School of Life and Medical Sciences, University of Hertfordshire, Hatfield AL10 9AB, UK; r.coutts@herts.ac.uk (R.H.A.C.); i.kotta-loizou2@herts.ac.uk (I.K.-L.); 3Hawkesbury Institute for the Environment, Western Sydney University, Richmond, NSW 2753, Australia; sam.el-kamand@westernsydney.edu.au; 4Department of Life Sciences, Faculty of Natural Sciences, Imperial College London, London SW7 2AZ, UK

## References

There was an error in the original publication [1]. We failed to mention the origin of the strain used in our publication. This correction requires the addition of the following reference:

27.Quesada-Moraga, E.; Vey, A. Intra-specific variation in virulence and in vitro production of macromolecular toxins active against locust among *Beauveria bassiana* strains and effects of in vivo and in vitro passage on these factors. *Biocontrol Sci. Technol.*
**2003**, *13*, 323–340.

A correction has been made to Section 2 Materials and Methods, 2.1. Maintenance of Fungal Isolates, Paragraph 1:

“This strain belongs to the culture collection of the Department of Agronomy of the University of Cordoba (Spain) and was originally isolated from infected specimens of the Moroccan locust *Dociostaurus maroccanus* (Thunberg) in the breeding area of La Serena in Badajoz (Spain). This strain has been deposited in the Spanish Type Culture Collection located at the University of Valencia under accession number CECT 20376 [27].”

## Corrected Paragraph


**2. Materials and Methods**



*2.1. Maintenance of Fungal Isolates*


This study relies on a *B. bassiana* isolate named “EABb 92/11-Dm” which is infected with two mycoviruses, *Beauveria bassiana* non-segmented virus (BbNV) 1 and Beauveria bassiana polymycovirus (BbPmV) 1. This strain belongs to the culture collection of the Department of Agronomy of the University of Cordoba (Spain) and was originally isolated from infected specimens of the Moroccan locust *Dociostaurus maroccanus* (Thunberg) in the breeding area of La Serena in Badajoz (Spain). This strain has been deposited in the Spanish Type Culture Collection located at the University of Valencia under accession number CECT 20376 [27]. The virus-infected and virus-free isogenic lines used were generated as reported previously [17] and are herein designated BbVI and BbVF, respectively. Two virus-free, commercial isolates, ATCC 74040 and GHA, were also used. All isolates were maintained on potato dextrose agar (PDA; Sigma-Aldrich, St. Louis, MO, USA) or Sabouraud dextrose agar (SBA; Sigma-Aldrich, St. Louis, MO, USA) at 25 °C and 80% relative humidity [28]. A cocktail of antibiotics (ampicillin, kanamycin, and streptomycin, each at a final concentration of 100 μg/mL) was used during the cultivation of all strains to prevent bacterial contamination. The use of antibiotics ensured that bacterial contaminants did not interfere with the growth and observations of the fungal strains, thereby maintaining the integrity and accuracy of the experimental results.

The authors state that the scientific conclusions are unaffected. This correction was approved by the Academic Editor. The original publication has also been updated.
