# Supplementary material for: Transcriptomic Analysis Reveals Molecular Mechanisms Underpinning Mycovirus-Mediated Hypervirulence in Beauveria bassiana Infecting Tenebrio molitor
Source: J Fungi (Basel). 2025 Jan 15;11(1):63. doi: 10.3390/jof11010063 (PMC11766762; doi:10.3390/jof11010063)
Supplement: Supplementary file 1 [file jof-11-00063-s001.zip › jof-2947936-supplementary.pdf]

(A)

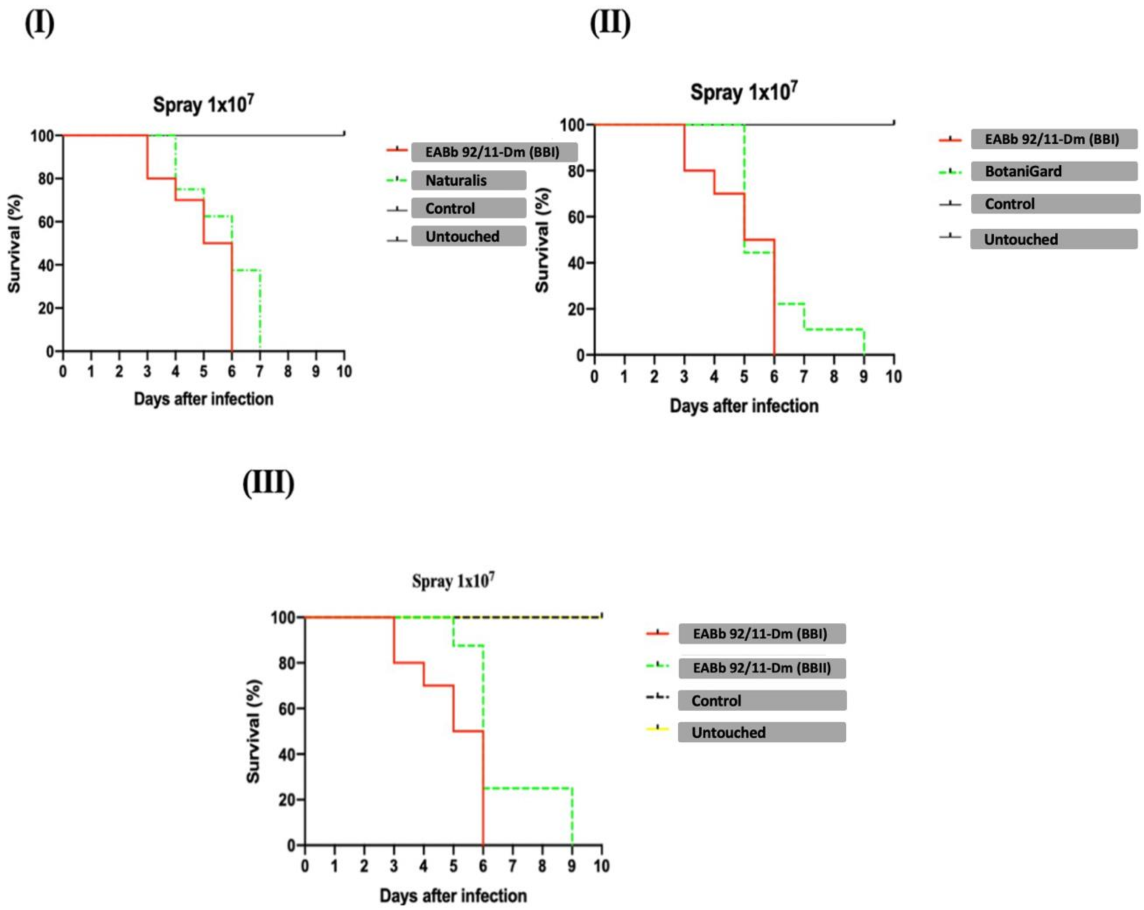
(I)
Spray 1x10⁷
Survival (%)
Days after infection
EABb 92/11-Dm (BBI)
Naturalis
Control
Untouched
(II)
Spray 1x10⁷
Survival (%)
Days after infection
EABb 92/11-Dm (BBI)
BotaniGard
Control
Untouched
(III)
Spray 1x10⁷
Survival (%)
Days after infection
EABb 92/11-Dm (BBI)
EABb 92/11-Dm (BBII)
Control
Untouched

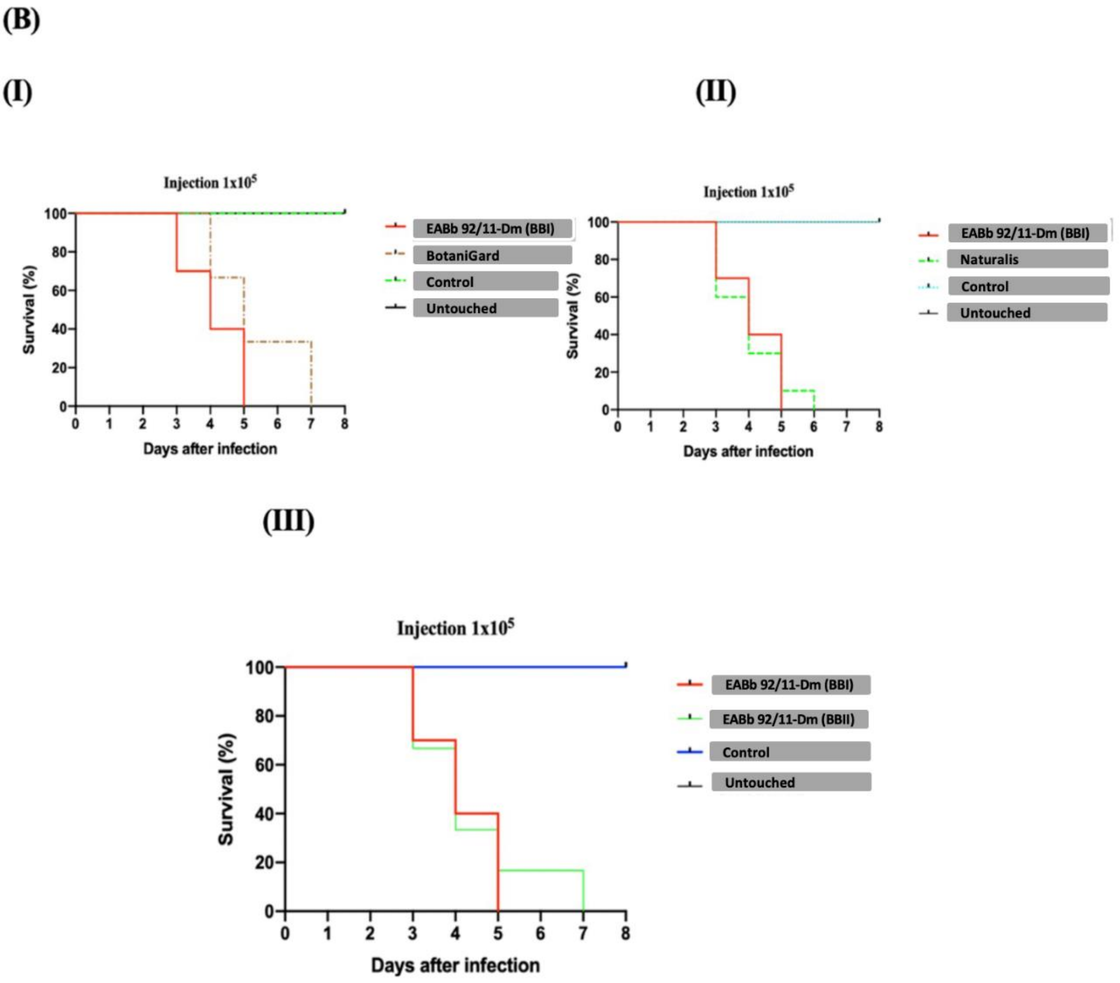

Figure S1: Survival curves of *Tenebrio molitor* larvae infected with *Beauveria bassiana* isolates following (A) topical application with 10^7 spores/larva or (B) direct injection with 10^5 spores/larva. Infections with *Beauveria bassiana* virus-infected (BbVI) and virus-free (BbVF) isogenic lines plus two commercial isolates Naturalis and BotaniGard were compared with control (insects sprayed or injected with phosphate buffer saline) and untouched (insects were neither sprayed nor injected). Mean survival of *T. molitor* larvae over a 9-day incubation period was plotted and survival curves were statistically analysed according to Kaplan-Meier estimation using the GraphPad Prism 8.0 software. P-values were estimated using Welch's t-test in Rstudio.

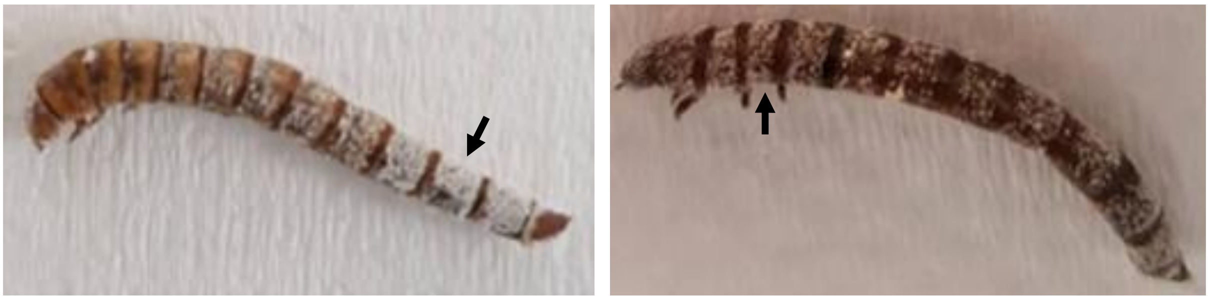

Figure S2: Mycosis (black arrows) was observed on the majority of the infected insects 72 h post-mortem. *T. molitor* larva inoculated with BbVI *via* topical application (left). *T. molitor* larva inoculated with BbVI *via* injection (right).

Table S1: Survival assay LT_{50} ± SE following topical application

***B. bassiana* isolate**	**LT_{50} ± SE**
EABb 92/11-Dm virus-infected (BbVI)	4.5 days ± 0.21
EABb 92/11-Dm virus-free (BbVF)	5.7 days ± 0.322
Naturalis	5.1 days ± 0.221
Botanigard	5.3 days ± 0.26

Table S2: Survival assay LT_{50} ± SE following injection

***B. bassiana* isolate**	**LT_{50} ± SE**
EABb 92/11-Dm virus-infected (BbVI)	3.5 days ± 0.2
EABb 92/11-Dm virus-free (BbVF)	4.7 days ± 0.26
Naturalis	3.8 days ± 0.23
Botanigard	3.9 days ± 0.28

Table S3: Manual curated alternative spliced genes.

Organism	Alternative spliced genes	Link
Beauveria bassiana	CUE domain-containing protein 5	https://curations.stressedfruitfly.com/apollo/annotator/loadLink?loc=373af319-180a-42af-afd2-8bcd9fae7029&organism=78822&tracks=OGS_2020,OGS_2021
Beauveria bassiana	Metal resistance protein YCF1	https://curations.stressedfruitfly.com/apollo/annotator/loadLink?loc=50369328-6548-405b-a5cf-6e845575a837&organism=78822&tracks=OGS_2020,OGS_2021
Beauveria bassiana	Tetratricopeptide-like protein	https://curations.stressedfruitfly.com/apollo/annotator/loadLink?loc=18ab957b-dcdc-44b2-a089-5b0d9ef410f4&organism=78822&tracks=OGS_2020,OGS_2021
Beauveria bassiana	Pre-mRNA-splicing factor prp46	https://curations.stressedfruitfly.com/apollo/annotator/loadLink?loc=08cf14ee-3cd9-4943-85bf-9d39cd8e8228&organism=78822&tracks=OGS_2020,OGS_2021
Beauveria bassiana	Nucleoporin nup44	https://curations.stressedfruitfly.com/apollo/annotator/loadLink?loc=001f4ef2-99e1-4d63-bde6-2bb54903d2f0&organism=78822&tracks=OGS_2020,OGS_2021
Beauveria bassiana	THO complex subunit 3	https://curations.stressedfruitfly.com/apollo/annotator/loadLink?loc=3e4fcf3c-2e75-4f95-992a-1b5b895e4373&organism=78822&tracks=OGS_2020,OGS_2021
Beauveria bassiana	Nucleoporin	https://curations.stressedfruitfly.com/apollo/annotator/loadLink?loc=5da1daf0-9f5c-4dd5-b712-aa3412bc3581&organism=78822&tracks=OGS_2020,OGS_2021
Beauveria bassiana	INO80 complex subunit 2	https://curations.stressedfruitfly.com/apollo/annotator/loadLink?loc=f6dab647-5cd6-4f6a-9b78-ee80f485d47f&organism=78822&tracks=OGS_2020,OGS_2021
Beauveria bassiana	2-isopropylmalate synthase	https://curations.stressedfruitfly.com/apollo/annotator/loadLink?loc=d5df1ba6-84cf-489c-a439-6083649b20ba&organism=78822&tracks=OGS_2020,OGS_2021
Beauveria bassiana	RING-box protein 1	https://curations.stressedfruitfly.com/apollo/annotator/loadLink?loc=9ccfe82b-a736-45d6-91b4-3cb3f0684f64&organism=78822&tracks=OGS_2020,OGS_2021
Beauveria bassiana	Transketolase	https://curations.stressedfruitfly.com/apollo/annotator/loadLink?loc=fd8ce806-1ebd-465f-b150-

		f1948eb504f6&organism=78822&tracks=OGS_2020,OGS_2021
Beauveria bassiana	Phosphatidylserine decarboxylase proenzyme 2	https://curations.stressedfruitfly.com/apollo/annotator/loadLink?loc=ebe6b6be-8426-4291-99d4-c50311a76d44&organism=78822&tracks=OGS_2020,OGS_2021
Beauveria bassiana	Elongin-C	https://curations.stressedfruitfly.com/apollo/annotator/loadLink?loc=4618d6d1-c073-40c5-9cd5-fe84b9d7db88&organism=78822&tracks=OGS_2020,OGS_2021
Beauveria bassiana	GTPase-activating protein GYP5	https://curations.stressedfruitfly.com/apollo/annotator/loadLink?loc=95ac0885-c3ba-455d-bb30-c2206ebd7b46&organism=78822&tracks=OGS_2020,OGS_2021
Beauveria bassiana	Casein kinase I hhp1	https://curations.stressedfruitfly.com/apollo/annotator/loadLink?loc=cedbc0a7-4aee-4b9b-8673-10d9be1256de&organism=78822&tracks=OGS_2020,OGS_2021
Beauveria bassiana	DNA damage checkpoint control protein rad1	https://curations.stressedfruitfly.com/apollo/annotator/loadLink?loc=5458c19c-76dc-41e8-bf57-76f3c44d9a32&organism=78822&tracks=OGS_2020,OGS_2021
Beauveria bassiana	Sir2 family protein	https://curations.stressedfruitfly.com/apollo/annotator/loadLink?loc=6be8d24a-1260-415b-9408-d092157b680e&organism=78822&tracks=OGS_2020,OGS_2021
Beauveria bassiana	DUF1253 domain-containing protein	https://curations.stressedfruitfly.com/apollo/annotator/loadLink?loc=222c5c30-af88-4785-964c-8e2f688ef16f&organism=78822&tracks=OGS_2020,OGS_2021
Beauveria bassiana	Meiotically up-regulated 72 protein	https://curations.stressedfruitfly.com/apollo/annotator/loadLink?loc=bc233f71-78a4-4cef-8a07-4264eb5c6db1&organism=78822&tracks=OGS_2020,OGS_2021
Beauveria bassiana	Cell division control protein 3	https://curations.stressedfruitfly.com/apollo/annotator/loadLink?loc=762e43c2-969c-495f-a092-fd2e58fa6683&organism=78822&tracks=OGS_2020,OGS_2021
Beauveria bassiana	NADH-ubiquinone oxidoreductase subunit, mitochondrial	https://curations.stressedfruitfly.com/apollo/annotator/loadLink?loc=ee43aeef-a599-4b6b-b7c0-b09519eb571f&organism=78822&tracks=OGS_2020,OGS_2021
Beauveria bassiana	Dolichyl-phosphate-mannose--protein	https://curations.stressedfruitfly.com/apollo/annotator/loadLink?loc=b06e4e61-ff3d-4b0e-b11e-42c3a5ed62dd&organism=78822&tracks=OGS_2020,OGS_2021

	mannosyltransferase 4	
Beauveria bassiana	Checkpoint kinase 2	https://curations.stressedfruitfly.com/apollo/annotator/loadLink?loc=8d6440d1-73e4-461f-86ae-f2e32fa8432b&organism=78822&tracks=OGS_2020,OGS_2021
Beauveria bassiana	Vacuolar protein sorting-associated protein 35	https://curations.stressedfruitfly.com/apollo/annotator/loadLink?loc=8fac1098-6d6b-4eb1-b154-237912b47993&organism=78822&tracks=OGS_2020,OGS_2021
Beauveria bassiana	Endoglucanase, putative	https://curations.stressedfruitfly.com/apollo/annotator/loadLink?loc=347eb325-921a-44ef-a634-6173cb485fd1&organism=78822&tracks=OGS_2020,OGS_2021
Beauveria bassiana	Short-chain dehydrogenase/reductase family protein	https://curations.stressedfruitfly.com/apollo/annotator/loadLink?loc=4b098214-8a4a-4ffd-941f-8a13f5627391&organism=78822&tracks=OGS_2020,OGS_2021
Beauveria bassiana	Hexokinase	https://curations.stressedfruitfly.com/apollo/annotator/loadLink?loc=e23ca859-b477-4a35-ab27-b9dc3a3a1fa4&organism=78822&tracks=OGS_2020,OGS_2021
Beauveria bassiana	Protein transport protein SEC31	https://curations.stressedfruitfly.com/apollo/annotator/loadLink?loc=aff20f32-e862-41b2-900c-947ef7daa55d&organism=78822&tracks=OGS_2020,OGS_2021
Beauveria bassiana	Regulator of nonsense transcripts 1	https://curations.stressedfruitfly.com/apollo/annotator/loadLink?loc=1afcb1a1-b216-49dc-98ae-d9f62dca634e&organism=78822&tracks=OGS_2020,OGS_2021
Beauveria bassiana	NADH:ubiquinone oxidoreductase 13.4kD subunit	https://curations.stressedfruitfly.com/apollo/annotator/loadLink?loc=fd508dad-bdf1-4e0a-a42e-4267028fde13&organism=78822&tracks=OGS_2020,OGS_2021
Beauveria bassiana	6-phosphogluconate dehydrogenase, decarboxylating 2	https://curations.stressedfruitfly.com/apollo/annotator/loadLink?loc=aedd0181-8f00-4aef-bca5-738220d0fe87&organism=78822&tracks=OGS_2020,OGS_2021
Beauveria bassiana	ZZ type zinc finger domain-containing protein	https://curations.stressedfruitfly.com/apollo/annotator/loadLink?loc=56473a18-d131-4749-af71-42b8af9382c5&organism=78822&tracks=OGS_2020,OGS_2021
Bauveria bassiana	Serine/threonine-protein kinase SSN3	https://curations.stressedfruitfly.com/apollo/annotator/loadLink?loc=85dfec8c-ccb5-4f57-8ab9-

		40a24b34a2f2&organism=78822&tracks=OGS_2020,OGS_2021
Beauveria bassiana	Mannan endo-1,6-alpha-mannosidase DCW1	https://curations.stressedfruitfly.com/apollo/annotator/loadLink?loc=7a0482a0-9833-4d02-9a47-034ac84395c7&organism=78822&tracks=OGS_2020,OGS_2021
Beauveria bassiana	T-complex protein 1 subunit zeta	https://curations.stressedfruitfly.com/apollo/annotator/loadLink?loc=24724d43-87e3-402e-8bae-d1192baf6fc2&organism=78822&tracks=OGS_2020,OGS_2021
Beauveria bassiana	Aldehyde dehydrogenase	https://curations.stressedfruitfly.com/apollo/annotator/loadLink?loc=e3f1cd51-97df-41c7-a33b-57ccf461fe34&organism=78822&tracks=OGS_2020,OGS_2021
Beauveria bassiana	alpha/beta superfamily hydrolase	https://curations.stressedfruitfly.com/apollo/annotator/loadLink?loc=34b042e5-0071-449a-b5a3-bac62482dbcc&organism=78822&tracks=OGS_2020,OGS_2021
Beauveria bassiana	Ubiquitin carboxyl-terminal hydrolase	https://curations.stressedfruitfly.com/apollo/annotator/loadLink?loc=c34212fa-869d-4ba0-8ebb-a24cd533b001&organism=78822&tracks=OGS_2020,OGS_2021
Beauveria bassiana	Importin subunit beta-1	https://curations.stressedfruitfly.com/apollo/annotator/loadLink?loc=45681c3b-c988-40e9-84a9-511a8e10ef4d&organism=78822&tracks=OGS_2020,OGS_2021
Beauveria bassiana	Guanine nucleotide-binding protein subunit alpha	https://curations.stressedfruitfly.com/apollo/annotator/loadLink?loc=b896a397-c875-43f6-a43c-f592b9d26065&organism=78822&tracks=OGS_2020,OGS_2021
Beauveria bassiana	cAMP-dependent protein kinase regulatory subunit	https://curations.stressedfruitfly.com/apollo/annotator/loadLink?loc=bf43b037-5d78-4047-afb5-32f931464f50&organism=78822&tracks=OGS_2020,OGS_2021
Beauveria bassiana	Glutamine:fructose-6-phosphate amidotransferase	https://curations.stressedfruitfly.com/apollo/annotator/loadLink?loc=fec4ad88-5bc2-4f45-8349-c13f71ad892a&organism=78822&tracks=OGS_2020,OGS_2021
Beauveria bassiana	Potassium/sodium efflux P-type ATPase	https://curations.stressedfruitfly.com/apollo/annotator/loadLink?loc=55152f3d-d0de-488e-9b71-b077ea181dc1&organism=78822&tracks=OGS_2020,OGS_2021
Beauveria bassiana	Ribosome biogenesis protein MAK21	https://curations.stressedfruitfly.com/apollo/annotator/loadLink?loc=5987271f-c7d2-4ea6-b35d-46a2db514e4f&organism=78822&tracks=OGS_2020,OGS_2021

Beauveria bassiana	Ferulic acid esterase (FaeA)	https://curations.stressedfruitfly.com/apollo/annotator/loadLink?loc=46055110-0ffa-4772-b179-ba444c437091&organism=78822&tracks=OGS_2020,OGS_2021
Beauveria bassiana	Nucleus export protein BRL1	https://curations.stressedfruitfly.com/apollo/annotator/loadLink?loc=b1380f03-1752-4847-9d4f-9d10cd9533d2&organism=78822&tracks=OGS_2020,OGS_2021
Beauveria bassiana	Concanavalin A-like lectin/glucanase	https://curations.stressedfruitfly.com/apollo/annotator/loadLink?loc=cc255469-5537-48ea-a0be-80d5eef7e44c&organism=78822&tracks=OGS_2020,OGS_2021
Beauveria bassiana	AP-1-like transcription factor	https://curations.stressedfruitfly.com/apollo/annotator/loadLink?loc=b40e676e-6b67-4f19-8d90-057d5da7f27e&organism=78822&tracks=OGS_2020,OGS_2021
Beauveria bassiana	Mitochondrial substrate carrier	https://curations.stressedfruitfly.com/apollo/annotator/loadLink?loc=a72eba48-8f69-4530-b54b-8e53dc45e8cb&organism=78822&tracks=OGS_2020,OGS_2021
Beauveria bassiana	SNF2 family domain-containing protein	https://curations.stressedfruitfly.com/apollo/annotator/loadLink?loc=86340de6-1089-49f3-a68b-c10dbdc0aac6&organism=78822&tracks=OGS_2020,OGS_2021
Beauveria bassiana	Vegetative cell wall protein gp1	https://curations.stressedfruitfly.com/apollo/annotator/loadLink?loc=9772d843-2f97-40aa-bda2-bb63bb2f71a5&organism=78822&tracks=OGS_2020,OGS_2021
Beauveria bassiana	Eukaryotic initiation factor 4E	https://curations.stressedfruitfly.com/apollo/annotator/loadLink?loc=814bb593-96bf-47dd-87b4-9d6426ce4205&organism=78822&tracks=OGS_2020,OGS_2021
Beauveria bassiana	Reticulocyte-binding protein 2 a	https://curations.stressedfruitfly.com/apollo/annotator/loadLink?loc=df949002-ca93-4a16-9144-bdb2ca919806&organism=78822&tracks=OGS_2020,OGS_2021
Beauveria bassiana	F-box domain-containing protein	https://curations.stressedfruitfly.com/apollo/annotator/loadLink?loc=40d29942-ba4f-41e7-9247-9a98f601c9ca&organism=78822&tracks=OGS_2020,OGS_2021
Beauveria bassiana	Adhesion regulating molecule	https://curations.stressedfruitfly.com/apollo/annotator/loadLink?loc=d05a6511-0ea3-4bb0-bdae-dfa41219fa98&organism=78822&tracks=OGS_2020,OGS_2021
Beauveria bassiana	Inner centromere protein-related protein pic1	https://curations.stressedfruitfly.com/apollo/annotator/loadLink?loc=4807f7d2-952c-4fc3-bffe-

		caa202703ca9&organism=78822&tracks=OGS_2020,OGS_2021
Beauveria bassiana	C6 finger domain protein	https://curations.stressedfruitfly.com/apollo/annotator/loadLink?loc=711715bf-aef3-42d5-be56-8414042772eb&organism=78822&tracks=OGS_2020,OGS_2021
Beauveria bassiana	Ubiquitin-protein ligase Sel1/Ubx2	https://curations.stressedfruitfly.com/apollo/annotator/loadLink?loc=cf89d2f9-4f79-4080-a5bd-893617bfcaf4&organism=78822&tracks=OGS_2020,OGS_2021
Beauveria bassiana	Calcium-transporting ATPase	https://curations.stressedfruitfly.com/apollo/annotator/loadLink?loc=8343198a-83f4-4c17-8395-54c5792aea5c&organism=78822&tracks=OGS_2020,OGS_2021
Beauveria bassiana	WD repeat domain-containing protein	https://curations.stressedfruitfly.com/apollo/annotator/loadLink?loc=507569b6-6b25-4583-b0e4-2c5b18f89d5d&organism=78822&tracks=OGS_2020,OGS_2021
Beauveria bassiana	Frequency clock protein	https://curations.stressedfruitfly.com/apollo/annotator/loadLink?loc=5e25c8e5-0995-4433-935f-3e0ce18bf1bc&organism=78822&tracks=OGS_2020,OGS_2021
Beauveria bassiana	Major facilitator superfamily transporter	https://curations.stressedfruitfly.com/apollo/annotator/loadLink?loc=c2a4a6d5-94a4-4cfe-93ed-da2353958af9&organism=78822&tracks=OGS_2020,OGS_2021
Beauveria bassiana	Serine/threonine-protein kinase KIN4	https://curations.stressedfruitfly.com/apollo/annotator/loadLink?loc=66a4ad77-a734-40f1-b983-5a5e92535517&organism=78822&tracks=OGS_2020,OGS_2021
Beauveria bassiana	Glycosyltransferase family 2	https://curations.stressedfruitfly.com/apollo/annotator/loadLink?loc=4776bc7c-9f0b-47a7-8d11-2a6694a4e888&organism=78822&tracks=OGS_2020,OGS_2021
Beauveria bassiana	Bbagt1	https://curations.stressedfruitfly.com/apollo/annotator/loadLink?loc=af37e6c8-8ec5-4e3d-9cce-11a6886c9195&organism=78822&tracks=OGS_2020,OGS_2021
Beauveria bassiana	FoabaA-like_protein	https://curations.stressedfruitfly.com/apollo/annotator/loadLink?loc=28e41d9b-05fa-4b57-b5f2-b67730cfd98b&organism=78822&tracks=OGS_2020,OGS_2021

Table S4: Survival of *Tenebrio molitor* post-treatment with fungal strains using injection method

Method	Fungal strain	Concentration (1x10^7)	Alive at 24 hrs	Alive at 48 hrs
Injection	BbVI	Conidia	3	-
Injection	BbVF	Conidia	3	-
Injection	Naturalis	Conidia	3	-
Injection	Botanigard	Conidia	3	-
PBS Control	-	-	All Alive	All Alive

The table presents the survival of insects at 24 and 48 hours after treatment with fungal strains (BbVI, BbVF, Naturalis, and Botanigard) via the injection method at a concentration of 1x10^7 conidia. The PBS control group, which remained untreated, showed no mortality during the observation period, indicating the specificity of fungal treatments. All fungal-treated groups exhibited complete mortality within 48 hours.

Table S5. Survival of *Tenebrio molitor* post-treatment with fungal strains using spray method

Method	Fungal Strain	Concentration (1x10^7)	Alive 24hr	Alive 48hr	Alive 72hr	Alive 96hr	Alive 120hr	Alive 144hr	Alive 168hr
Spraying	BbVI	Conidia	10	10	9	3	1	-	-
Spraying	BbVF	Conidia	10	10	9	7	6	3	-
Spraying	Naturalis	Conidia	10	10	9	3	1	-	-
Spraying	Botanigard	Conidia	10	10	9	3	1	-	-
**PBS control**	-	-	All Alive	All Alive	All Alive	All Alive	All Alive	All Alive	All Alive

The table illustrates the survival of insects over time (24 to 168 hours) following treatment with with fungal strains (BbVI, BbVF, Naturalis, and Botanigard) using the spraying method. The concentrations of fungal conidia were 1x10^7 for BbVI, BbVF, Naturalis, and Botanigard, while the PBS control group remained untreated. The survival count decreases over time for fungal-treated groups, indicating fungal efficacy, whereas all individuals in the PBS control group survived throughout the observation period.

Table S6: Species identification, indicating Beauveria bassiana RNA Virus 1 only in BbVI at 21 dpi

SampleName	TotalReads	OverallAlignment (ASM28067) %	TotalSequencesClassifiedByKraken(%; db=fungi_refseq_latest)	TopHit (fungi_refseq_latest)	SequencesClassifiedAsTopHit(%; fungi_refseq_latest)	NextBestHit (fungi_refseq_latest)	SequencesClassifiedAsNextBestHit(%; fungi_refseq_latest)	TotalSequencesClassifiedByKraken(%; db=nt)	TopHit (nt)	SequencesClassifiedAsTopHit(%)	NextBestHit (nt)	SequencesClassifiedAsNextBestHit(%; nt)
subset3/BBVI-Day21-1_S24_L001_R1_001.fa	2456079	82.08	82.39	Beauveria ba	75.54	Cordyceps m	3.26	0.89	Beauveria bassiana RNA virus 1	0.4	Beauveria ba	0.15
subset3/BBVI-Day21-1_S24_L002_R1_001.fa	2411763	82.09	82.44	Beauveria ba	75.6	Cordyceps m	3.28	0.89	Beauveria bassiana RNA virus 1	0.4	Beauveria ba	0.15
subset3/BBVI-Day21-1_S24_L003_R1_001.fa	2432041	82.15	82.44	Beauveria ba	75.63	Cordyceps m	3.27	0.88	Beauveria bassiana RNA virus 1	0.41	Beauveria ba	0.15
subset3/BBVI-Day21-1_S24_L004_R1_001.fa	2415817	82.28	82.67	Beauveria ba	75.82	Cordyceps m	3.28	0.88	Beauveria bassiana RNA virus 1	0.39	Beauveria ba	0.16
subset3/BBVI-Day21-2_S25_L001_R1_001.fa	3362255	80.99	82.56	Beauveria ba	74.36	Cordyceps m	3.7	0.89	Beauveria bassiana RNA virus 1	0.44	Beauveria ba	0.15
subset3/BBVI-Day21-2_S25_L002_R1_001.fa	3312822	80.92	82.47	Beauveria ba	74.27	Cordyceps m	3.71	0.88	Beauveria bassiana RNA virus 1	0.44	Beauveria ba	0.14
subset3/BBVI-Day21-2_S25_L003_R1_001.fa	3324975	80.98	82.55	Beauveria ba	74.34	Cordyceps m	3.72	0.89	Beauveria bassiana RNA virus 1	0.45	Beauveria ba	0.15
subset3/BBVI-Day21-2_S25_L004_R1_001.fa	3302963	81.1	82.72	Beauveria ba	74.53	Cordyceps m	3.71	0.89	Beauveria bassiana RNA virus 1	0.45	Beauveria ba	0.14
subset3/BBVI-Day21-3_S26_L001_R1_001.fa	2285719	80.77	82.61	Beauveria ba	73.79	Cordyceps m	3.7	0.9	Beauveria bassiana RNA virus 1	0.41	Beauveria ba	0.16
subset3/BBVI-Day21-3_S26_L002_R1_001.fa	2245819	80.69	82.57	Beauveria ba	73.77	Cordyceps m	3.7	0.92	Beauveria bassiana RNA virus 1	0.41	Beauveria ba	0.17
subset3/BBVI-Day21-3_S26_L003_R1_001.fa	2261011	80.78	82.59	Beauveria ba	73.76	Cordyceps m	3.7	0.92	Beauveria bassiana RNA virus 1	0.42	Beauveria ba	0.16
subset3/BBVF-Day21-1_S27_L001_R1_001.fa	2322984	82.38	79.25	Beauveria ba	68.73	Cordyceps m	5.05	0.47	Beauveria bassiana	0.16	Acanthaster	0.04
subset3/BBVF-Day21-1_S27_L002_R1_001.fa	2275756	82.26	79.11	Beauveria ba	68.57	Cordyceps m	5.08	0.49	Beauveria bassiana	0.16	Acanthaster	0.04
subset3/BBVF-Day21-1_S27_L003_R1_001.fa	2297837	82.36	79.21	Beauveria ba	68.65	Cordyceps m	5.08	0.48	Beauveria bassiana	0.16	Acanthaster	0.04
subset3/BBVF-Day21-1_S27_L004_R1_001.fa	2274436	82.51	79.45	Beauveria ba	68.97	Cordyceps m	5.05	0.48	Beauveria bassiana	0.16	Acanthaster	0.04
subset3/BBVF-Day21-2_S28_L001_R1_001.fa	1650511	83.41	78.83	Beauveria ba	69.68	Cordyceps m	4.93	0.52	Beauveria bassiana	0.17	Acanthaster	0.06
subset3/BBVF-Day21-2_S28_L002_R1_001.fa	1624859	83.33	78.8	Beauveria ba	69.63	Cordyceps m	4.92	0.51	Beauveria bassiana	0.17	Acanthaster	0.06
subset3/BBVF-Day21-2_S28_L003_R1_001.fa	1633366	83.39	78.8	Beauveria ba	69.63	Cordyceps m	4.9	0.51	Beauveria bassiana	0.17	Acanthaster	0.06
subset3/BBVF-Day21-2_S28_L004_R1_001.fa	1623917	83.53	79.08	Beauveria ba	69.95	Cordyceps m	4.9	0.5	Beauveria bassiana	0.16	Acanthaster	0.06
subset3/BBVF-Day21-3_S29_L001_R1_001.fa	2993887	80.32	79.55	Beauveria ba	67.28	Cordyceps m	6.63	0.53	Beauveria bassiana	0.17	Acanthaster	0.06
subset3/BBVF-Day21-3_S29_L002_R1_001.fa	2934733	80.26	79.46	Beauveria ba	67.21	Cordyceps m	6.62	0.53	Beauveria bassiana	0.17	Acanthaster	0.06
subset3/BBVF-Day21-3_S29_L003_R1_001.fa	2960865	80.32	79.48	Beauveria ba	67.23	Cordyceps m	6.63	0.53	Beauveria bassiana	0.17	Acanthaster	0.06
subset3/BBVF-Day21-3_S29_L004_R1_001.fa	2935209	80.49	79.76	Beauveria ba	67.53	Cordyceps m	6.6	0.52	Beauveria bassiana	0.17	Acanthaster	0.07

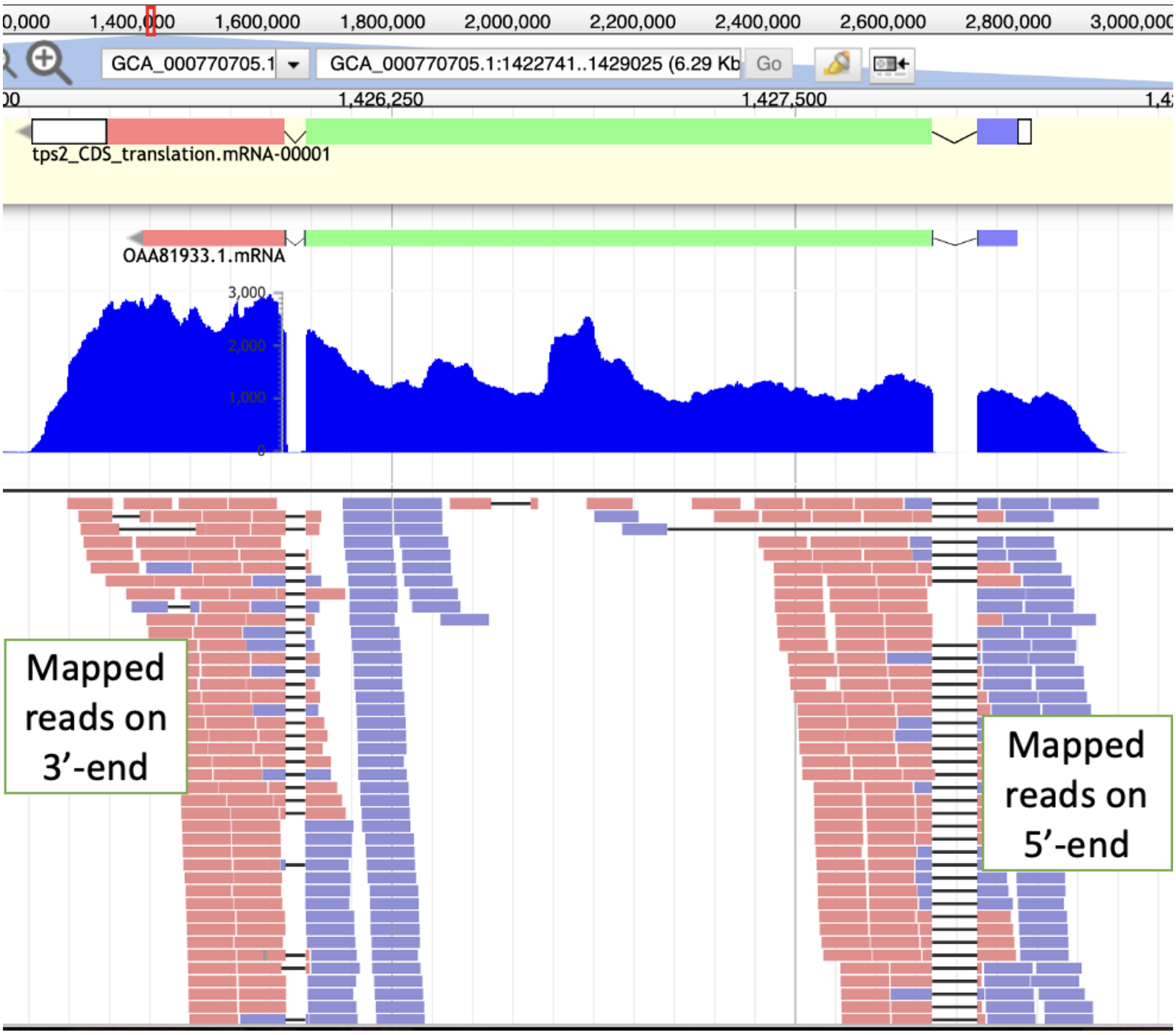

Figure S3: Manual curation of the 5' and 3' untranslated regions (UTRs) of the TPS2 gene. The white boxes represent the UTRs that were allocated using evidence such as "public RNA coverage" and "intron/exon junction reads".

Link access to the WebApollo curation site:

https://curations.stressedfruitfly.com/apollo/77156/jbrowse/index.html?loc=GCA_000770705.1:1424586..1429497&tracks=VirulenceFactors,JunctionReads,Public%20RNASeq%20coverage

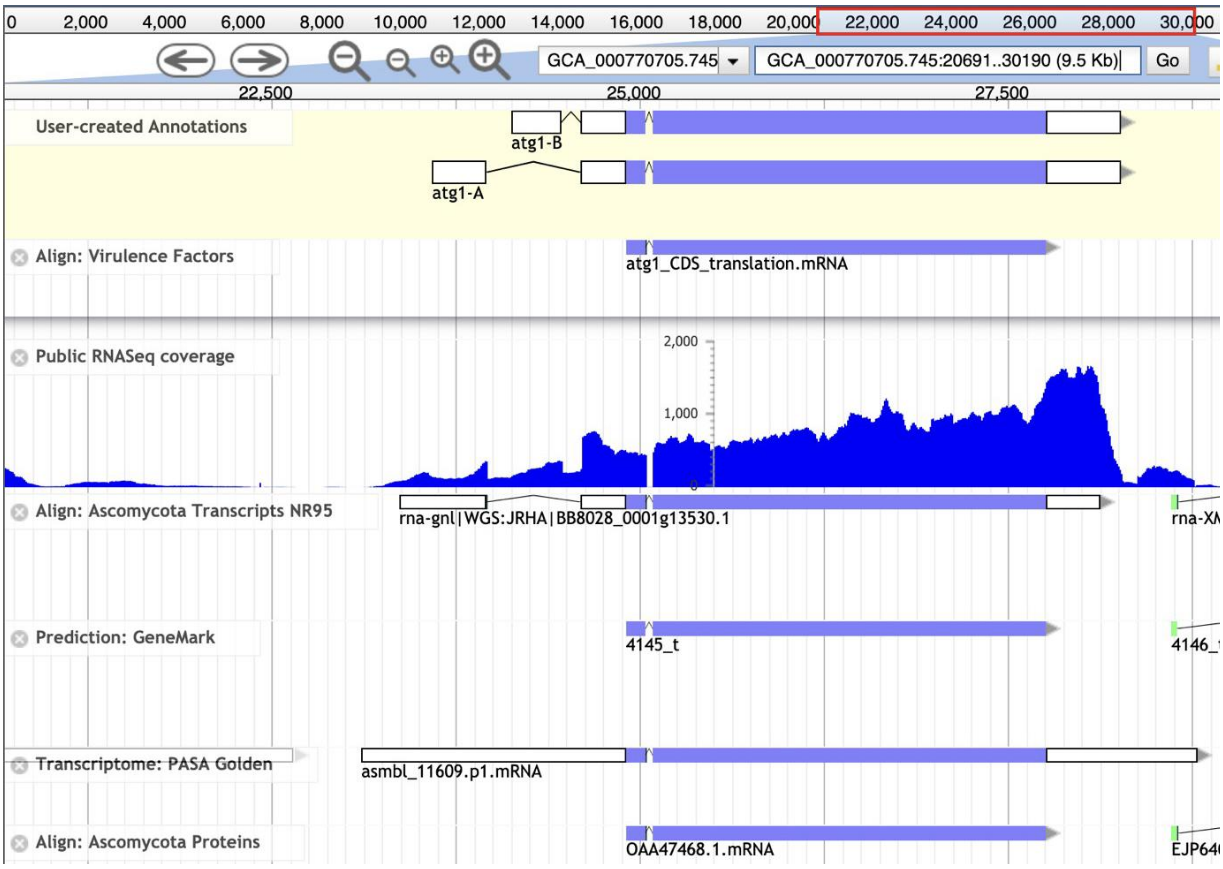

Figure S4: Manual curation of an alternatively spliced isoform in the ATG1 gene. The Apollo editing window shows a "user-created annotation" at the top, followed by incorrect atg1_CDS_translation.mRNA ("align: virulence factors"). The "public RNA-seq coverage" illustrates the RNA coverage, which was used to allocate the UTRs, and is followed by outcomes of gene prediction software such as GeneMark, Augustus, and PASA. Interestingly, none of these suggested the alternatively spliced isoform.

Link access to the WebApollo/JBrowser curation site:

https://curations.stressedfruitfly.com/apollo/77156/jbrowse/index.html?loc=GCA_000770705.745:20690..30190&tracks=AscomycotaProteins,VirulenceFactors,JunctionReads,Public%20RNASeq%20coverage,AscomycotaTranscriptsNr95,GeneMarkPred,PASAGOLDEN

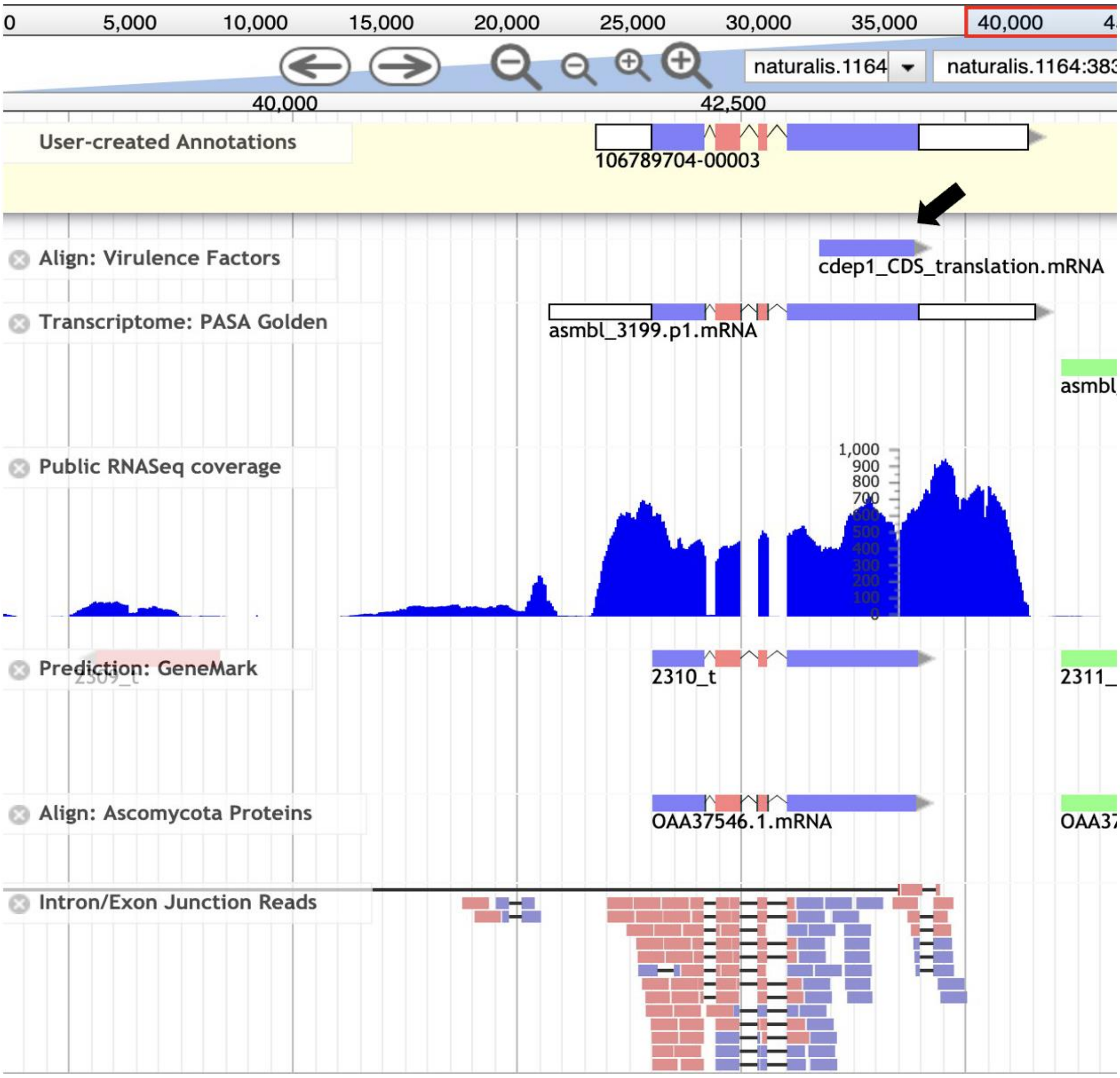

Figure S5: Manual curation of introns and exons in the CDEP1 gene. The CDEP1 gene first appeared as a single exon ("align: virulence factors") before manual curation as indicated by the black arrow. Following evidence provided from "public RNA coverage" and intron/exon junction reads, the correct final annotation was reached and indicated as "user created annotation".

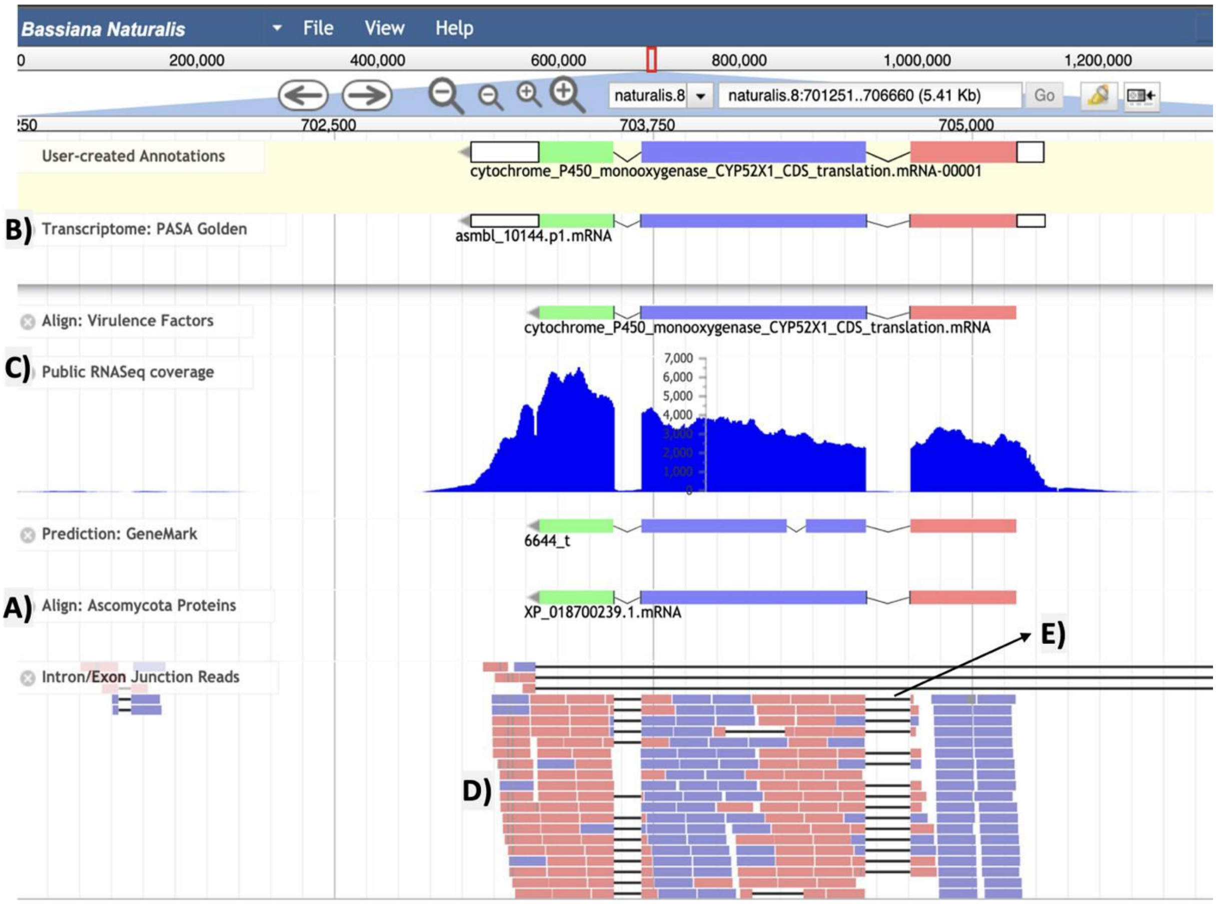

Figure S6: Examples of updated curation in Naturalis. The curated gene model is compared to (A) to Ascomycota proteins, (B) PASA Golden refinement, (C) public RNA-seq coverage, and (D) aligned reads; (E) intronic regions are shown by lines and white boxes represent UTRs. Link access to the WebApollo/JBrowser curation site: https://curations.stressedfruitfly.com/apollo/77156/jbrowse/index.html?loc=GCA_000770705.8:664543..667106&tracks=VirulenceFactors,JunctionReads,Public%20RNASeq%20coverage

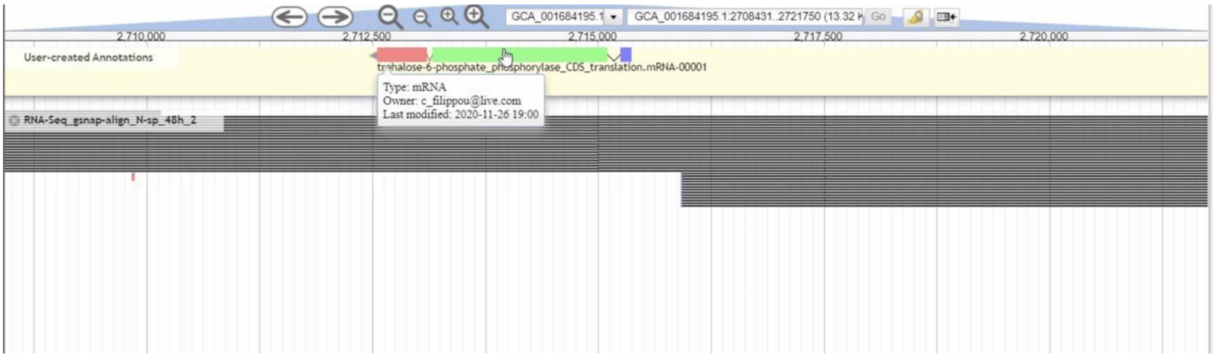

Figure S7: Spurious alignments before Burrows-Wheeler Aligner (BWA) mapping alignment.

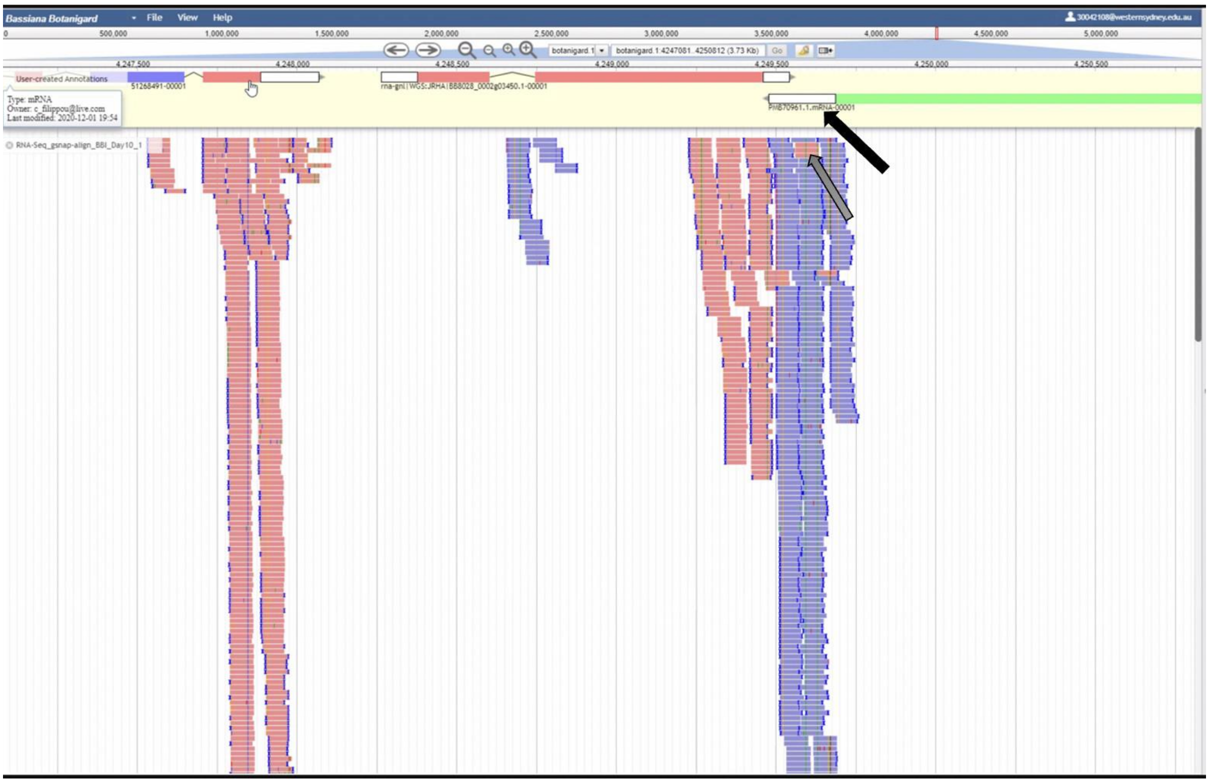

Figure S8: The black arrow indicates the correct coverage of the 3' UTR after final curation. The grey arrow indicates spurious alignments (the reads are outside the predicted 3' UTR coverage).

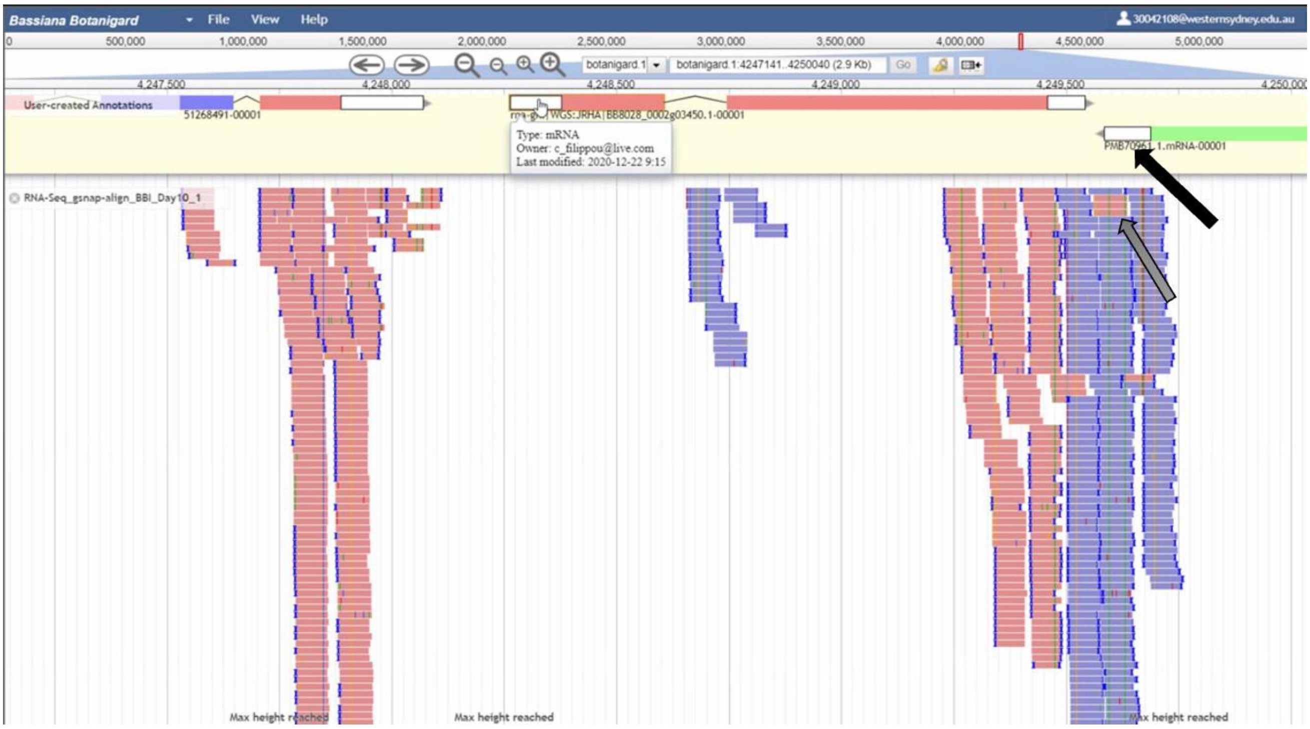

Figure S9: Black arrow indicates incorrect coverage of the 3' UTR after final curation. Grey arrow indicates spurious alignments (the reads are outside the predicted 3' UTR coverage).

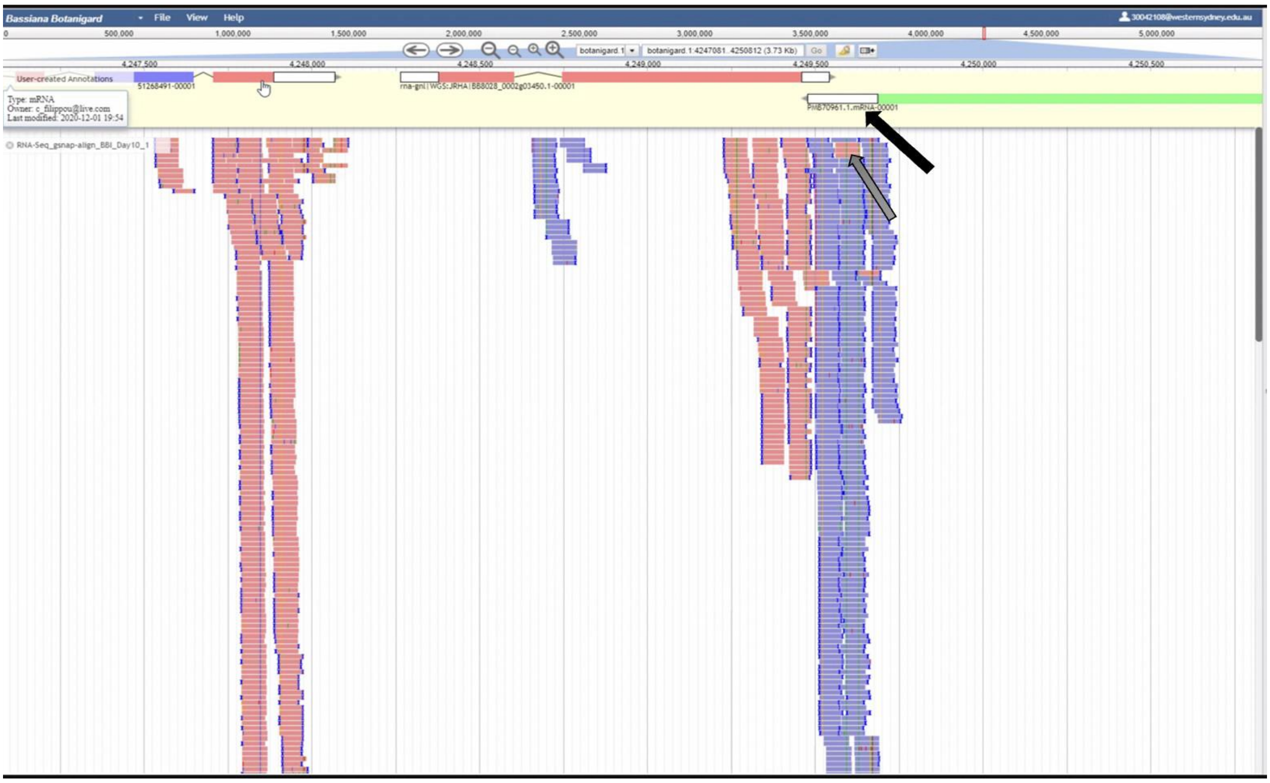

Figure S10: The black arrow indicates the correct coverage of the 3' UTR after final curation. The grey arrow indicates spurious alignments (the reads are outside the predicted 3' UTR coverage).

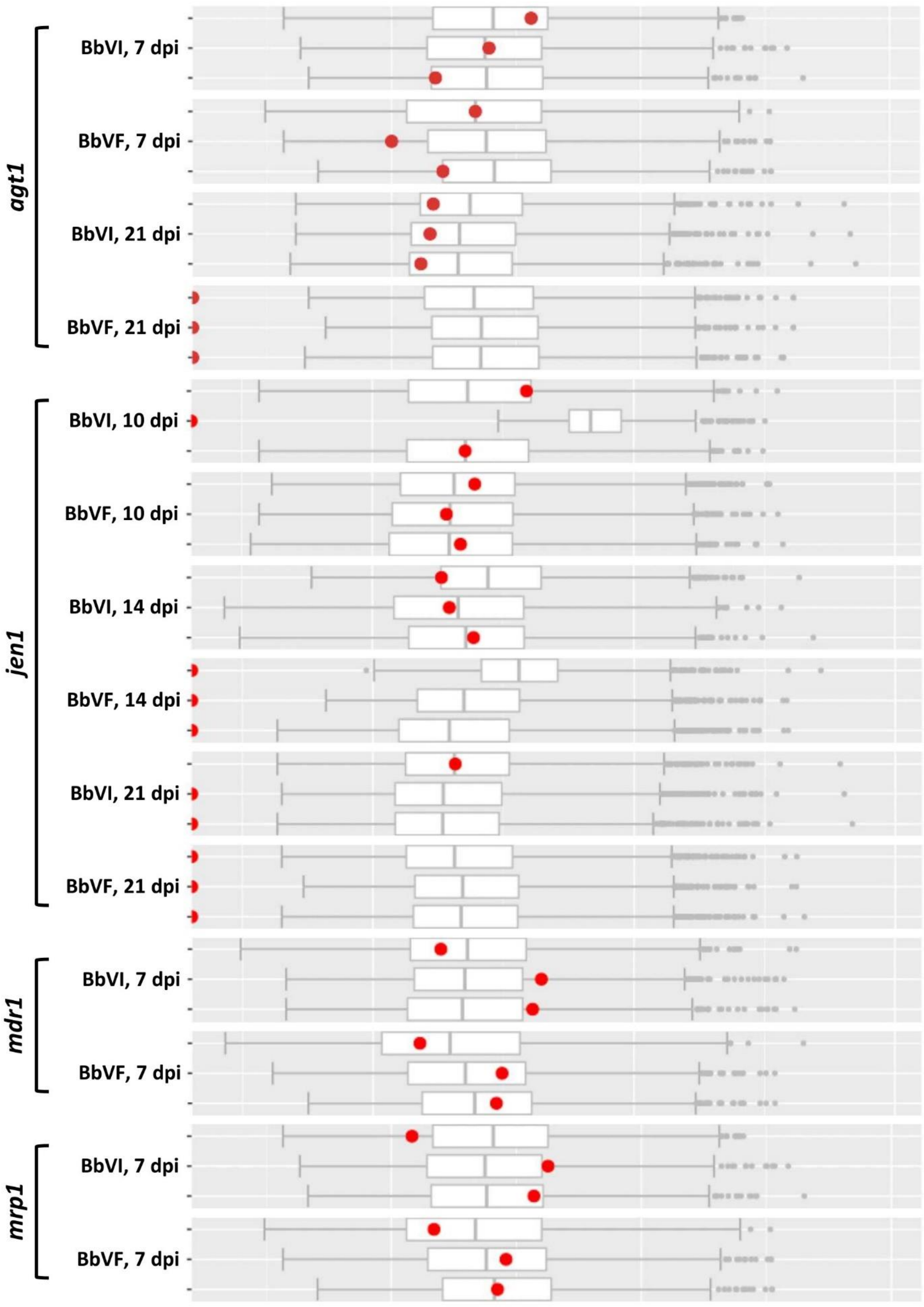

Figure S11: Boxplots demonstrating the expression profiles of fungal transporter genes *agt1*, *jen1*, *mdr1* and *mrp1 in vitro*.

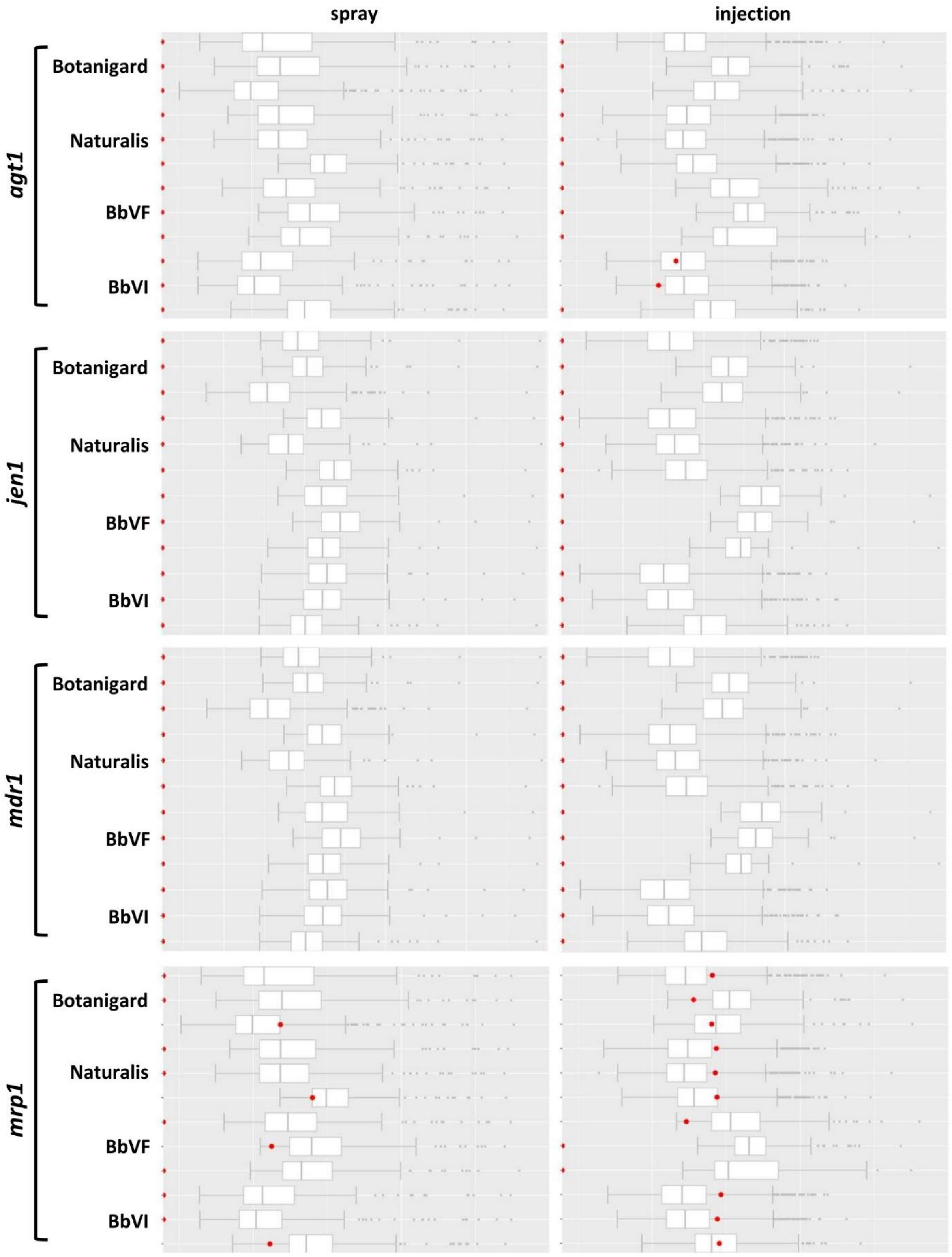

Figure S12: Boxplots demonstrating the expression profiles of fungal transporter genes *agt1*, *jen1*, *mdr1* and *mrp1* following infection of *T. molitor* at 48 dpi.

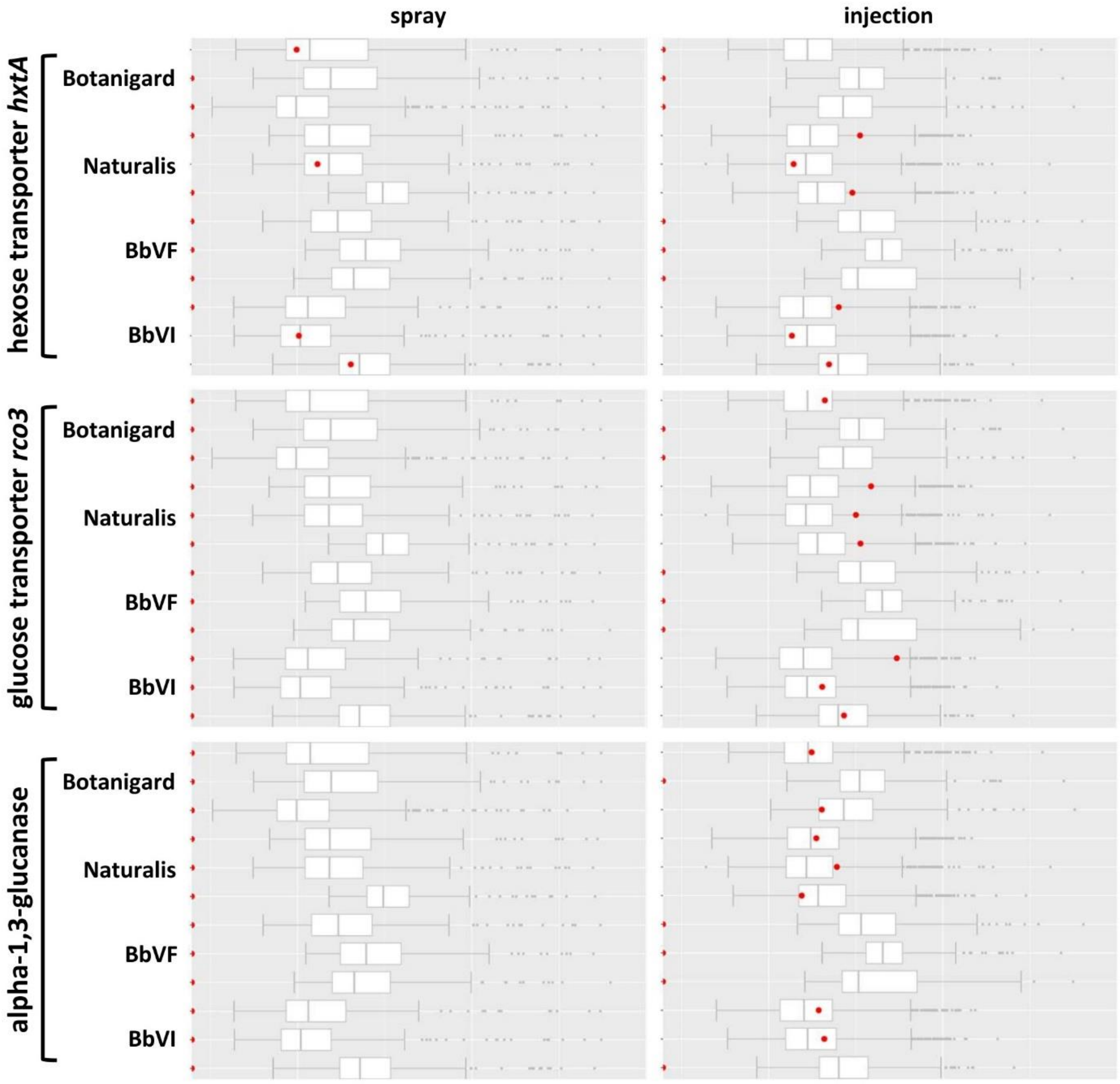

Figure S13: Boxplots demonstrating the expression profiles of fungal hexose transporter *hxtA*, glucose transporter *rco3* and alpha-1,3-glucanase genes following infection of *T. molitor* at 48 dpi.

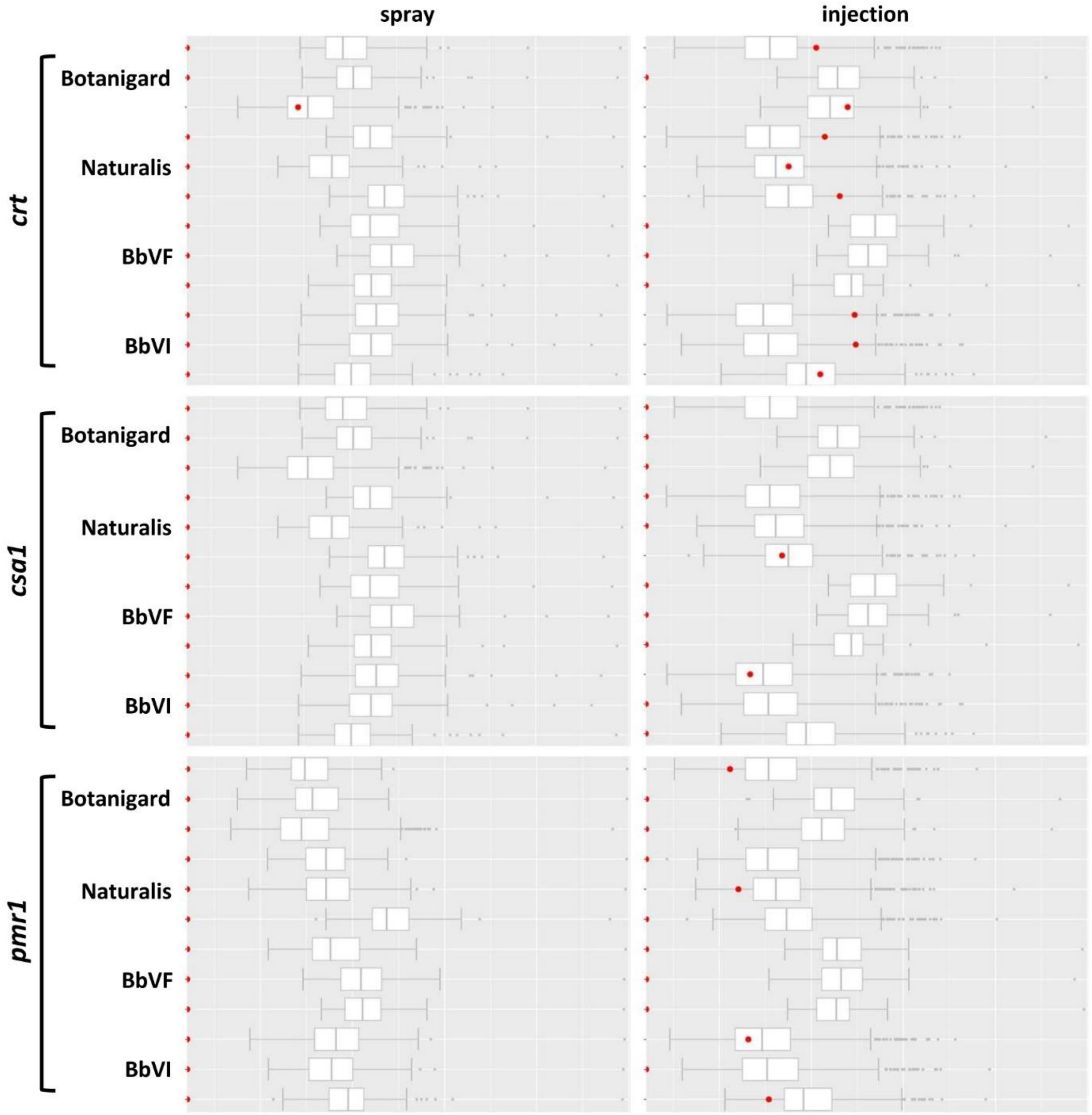

Figure S14: Boxplots demonstrating the expression profiles of fungal *crt*, *csa1* and *pmr1* genes following infection of *T. molitor* at 48 dpi.

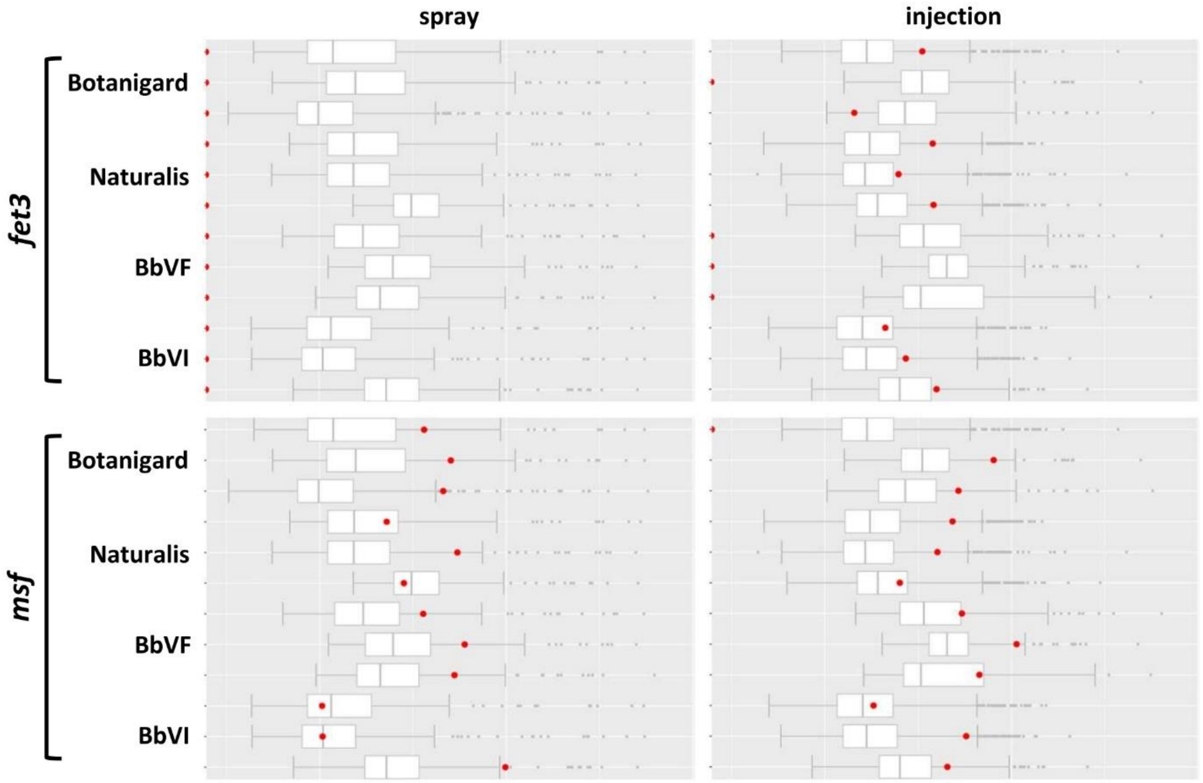

Figure S15: Boxplots demonstrating the expression profiles of fungal *fet3* and *mfs* genes following infection of *T. molitor* at 48 dpi.

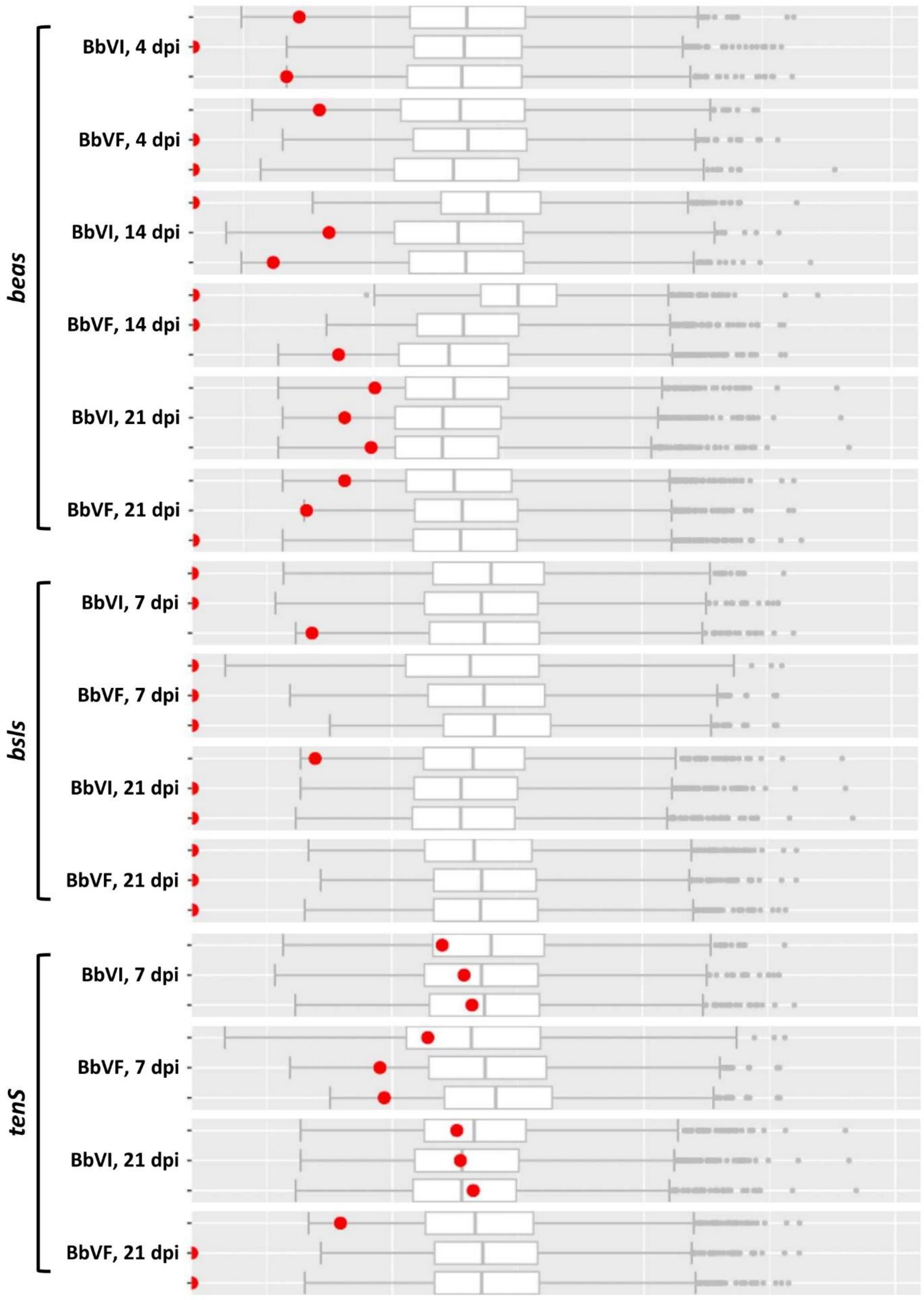

Figure S16: Boxplots demonstrating the expression profiles of fungal mycotoxin genes *beas*, *bsls* and *tenS in vitro*.

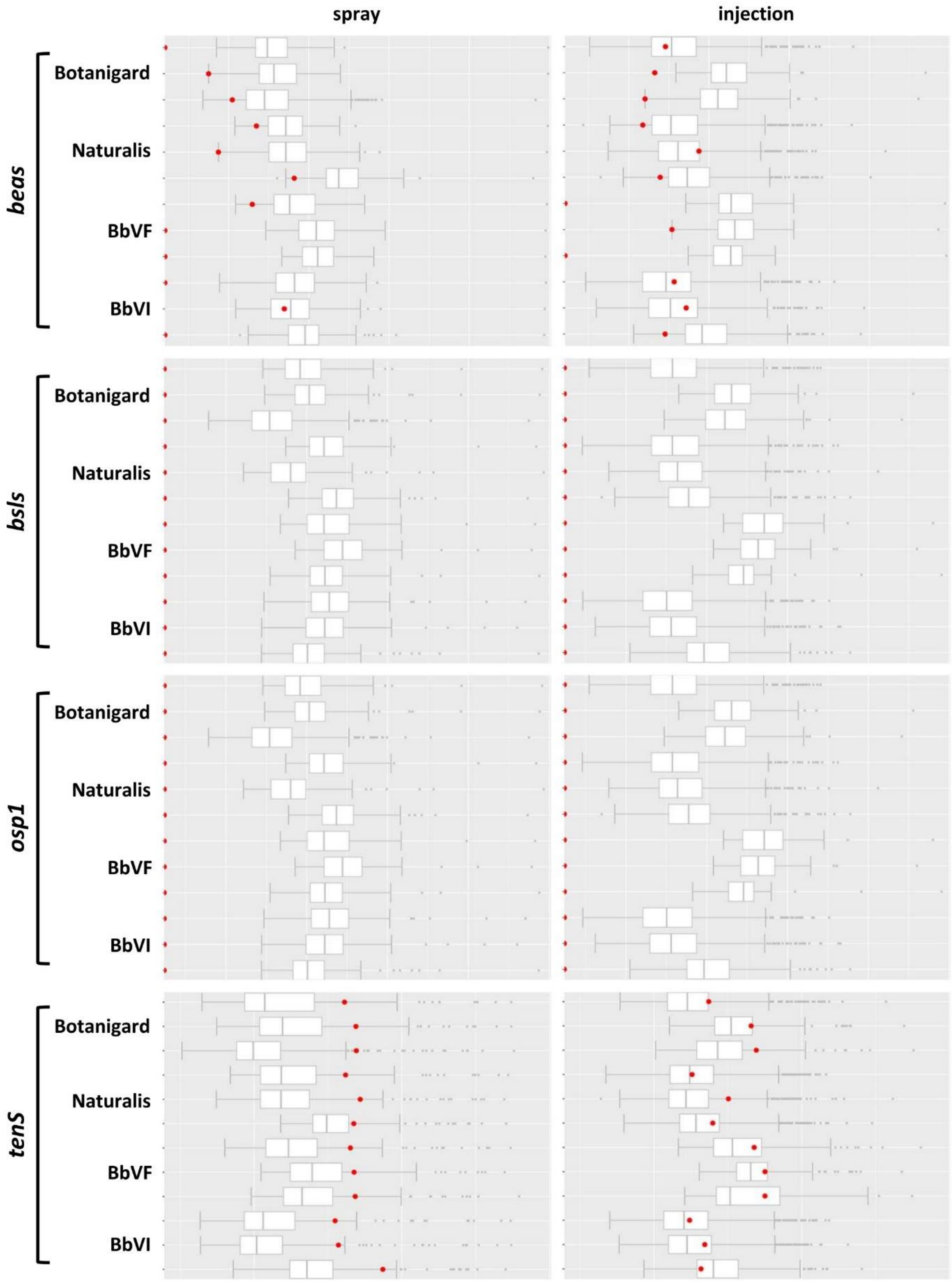

Figure S17: Boxplots demonstrating the expression profiles of fungal mycotoxin genes *beas*, *bsls*, *osp1* and *tenS* following infection of *T. molitor* at 48 dpi.

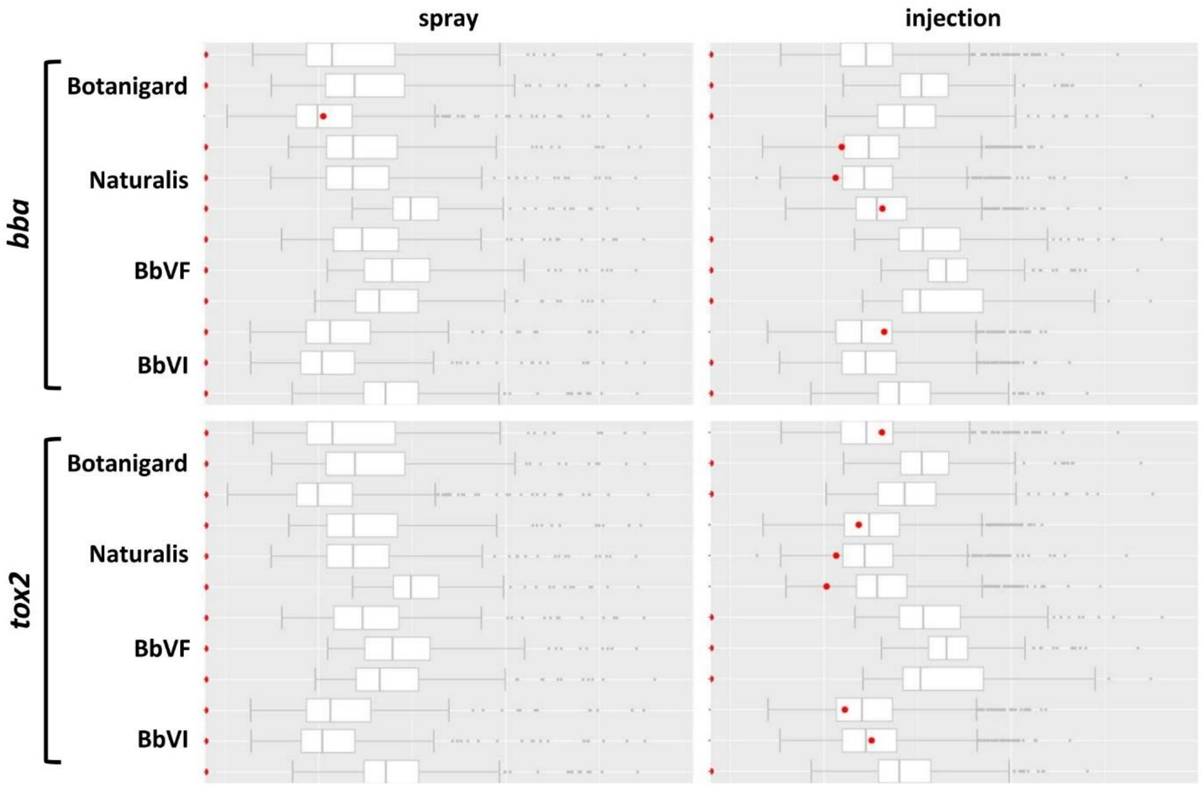

Figure S18: Boxplots demonstrating the expression profiles of fungal mycotoxin genes *bba* and *tox2* following infection of *T. molitor* at 48 dpi.

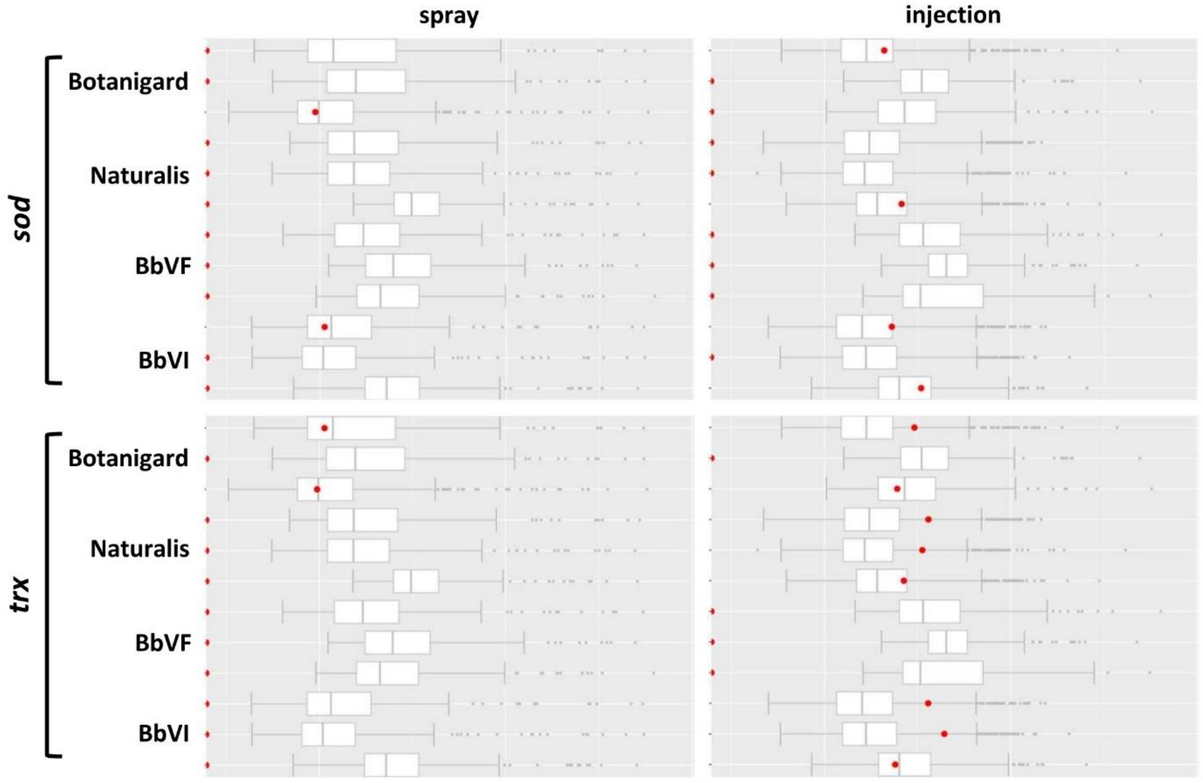

Figure S19: Boxplots demonstrating the expression profiles of fungal oxidative stress genes *sod* and *trx* following infection of *T. molitor* at 48 dpi.

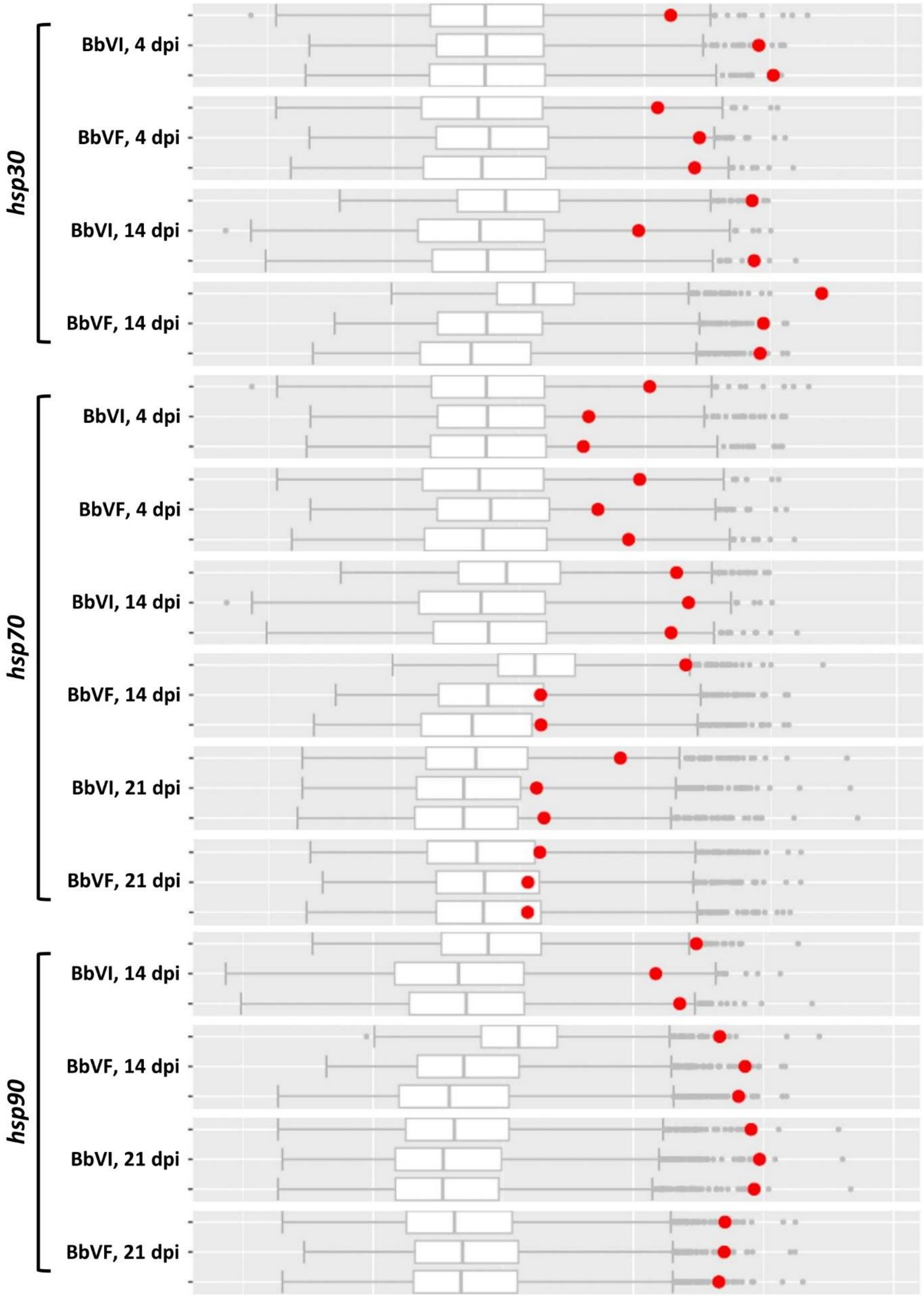

Figure S20: Boxplots demonstrating the expression profiles of fungal heat shock protein genes *hsp30*, *hsp70* and *hsp90 in vitro*.

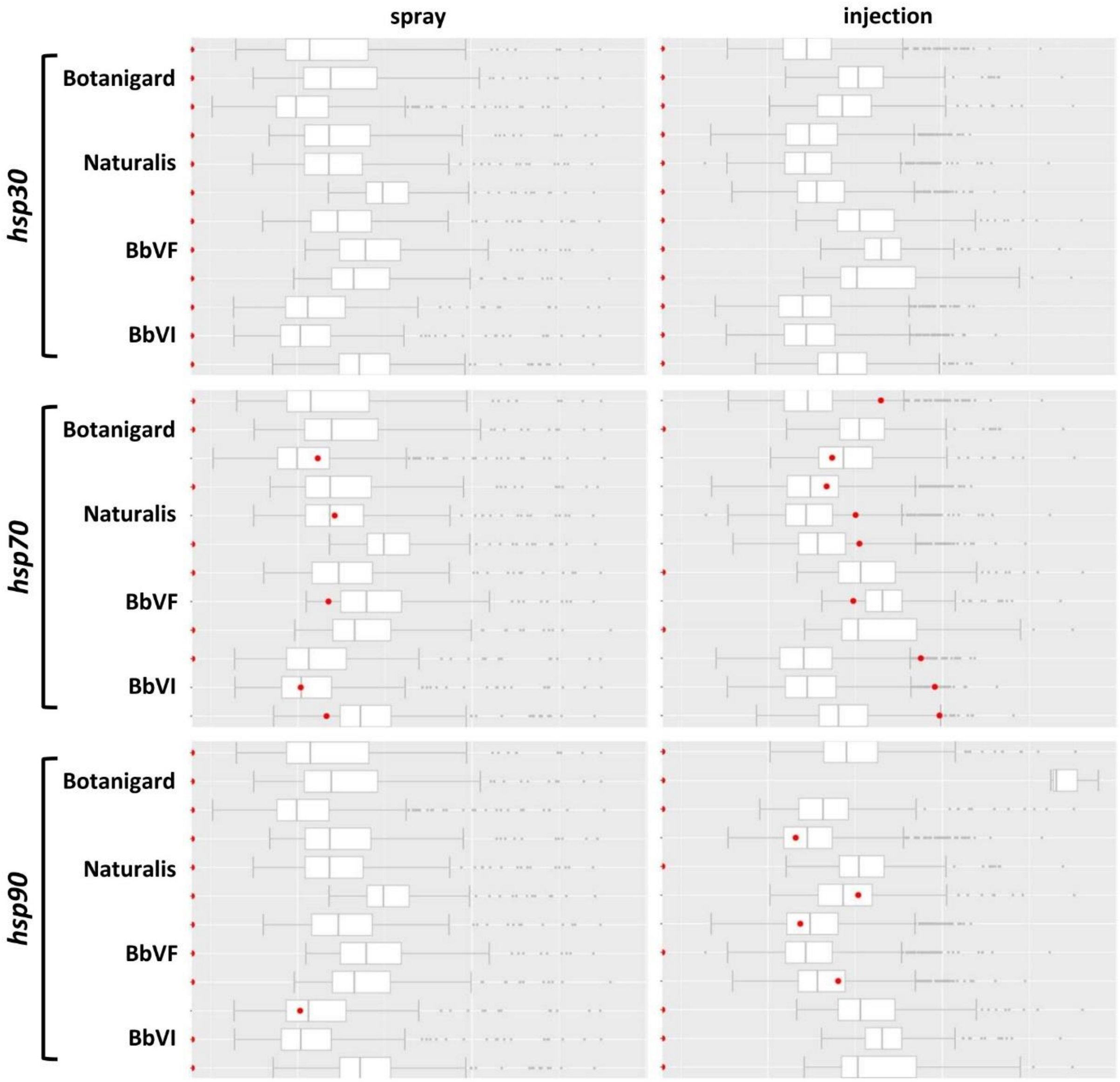

Figure S21: Boxplots demonstrating the expression profiles of fungal heat shock protein genes *hsp30*, *hsp70* and *hsp90* following infection of *T. molitor* at 48 dpi.

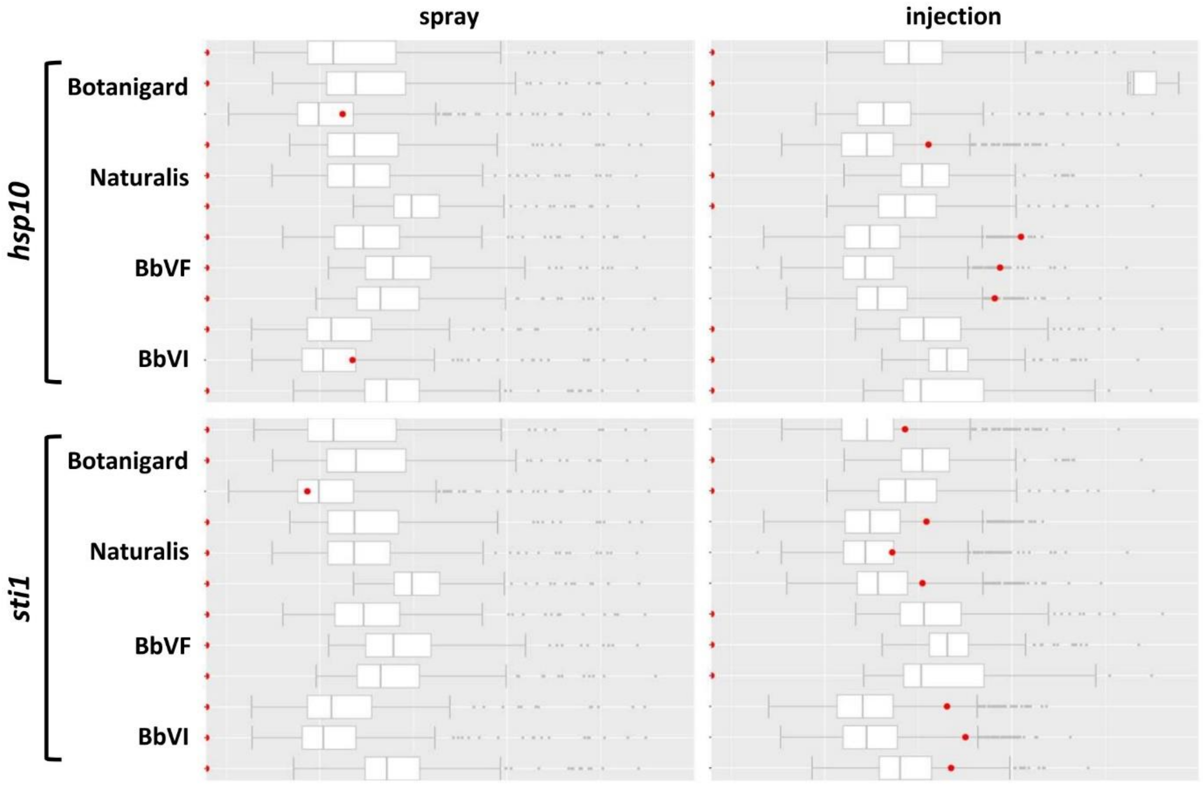

Figure S22: Boxplots demonstrating the expression profiles of fungal heat shock protein genes *hsp10* and *sti1* following infection of *T. molitor* at 48 dpi.

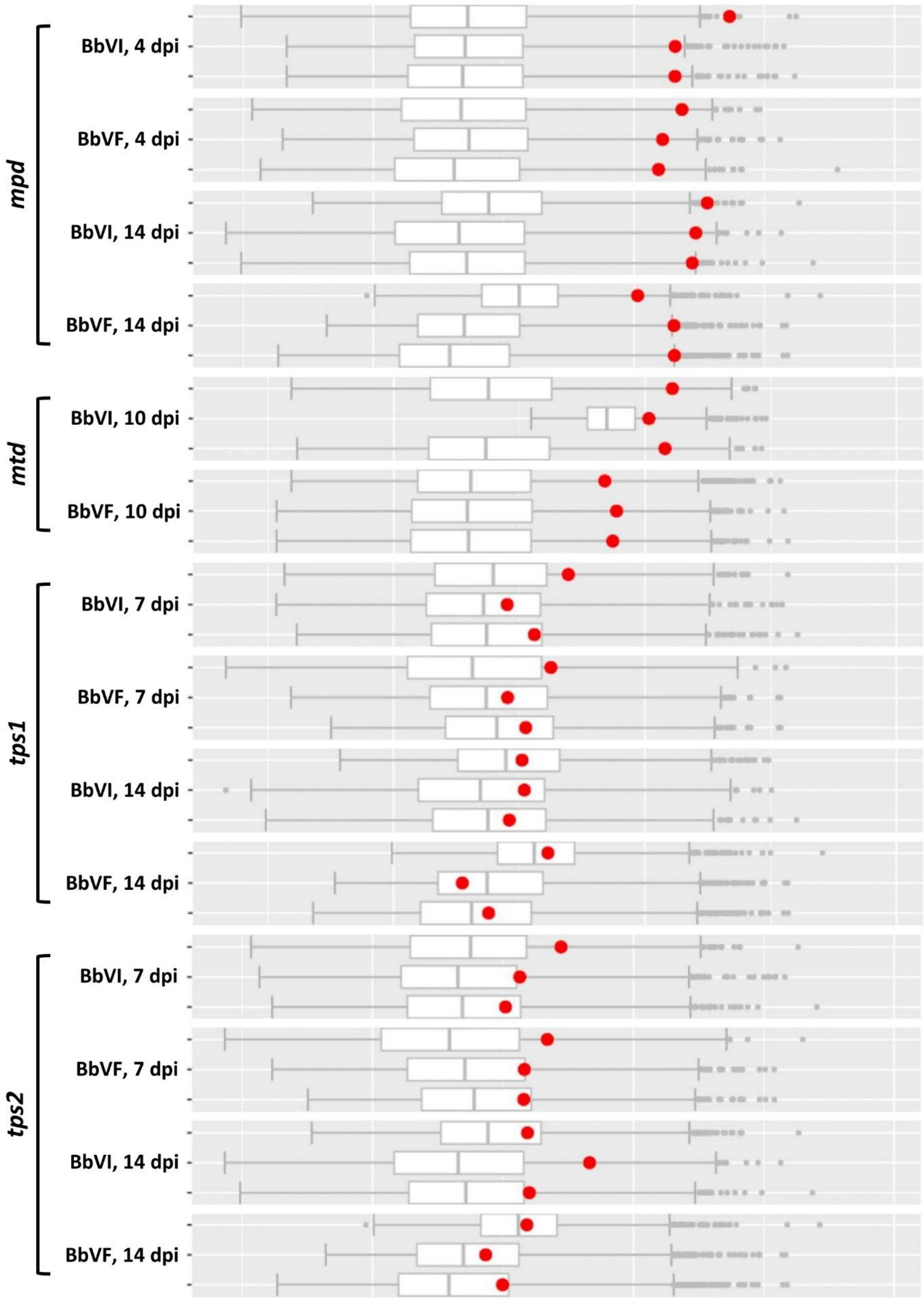

Figure S23: Boxplots demonstrating the expression profiles of fungal metabolism genes *mpd*, *mtd*, *tps1* and *tps2 in vitro*.

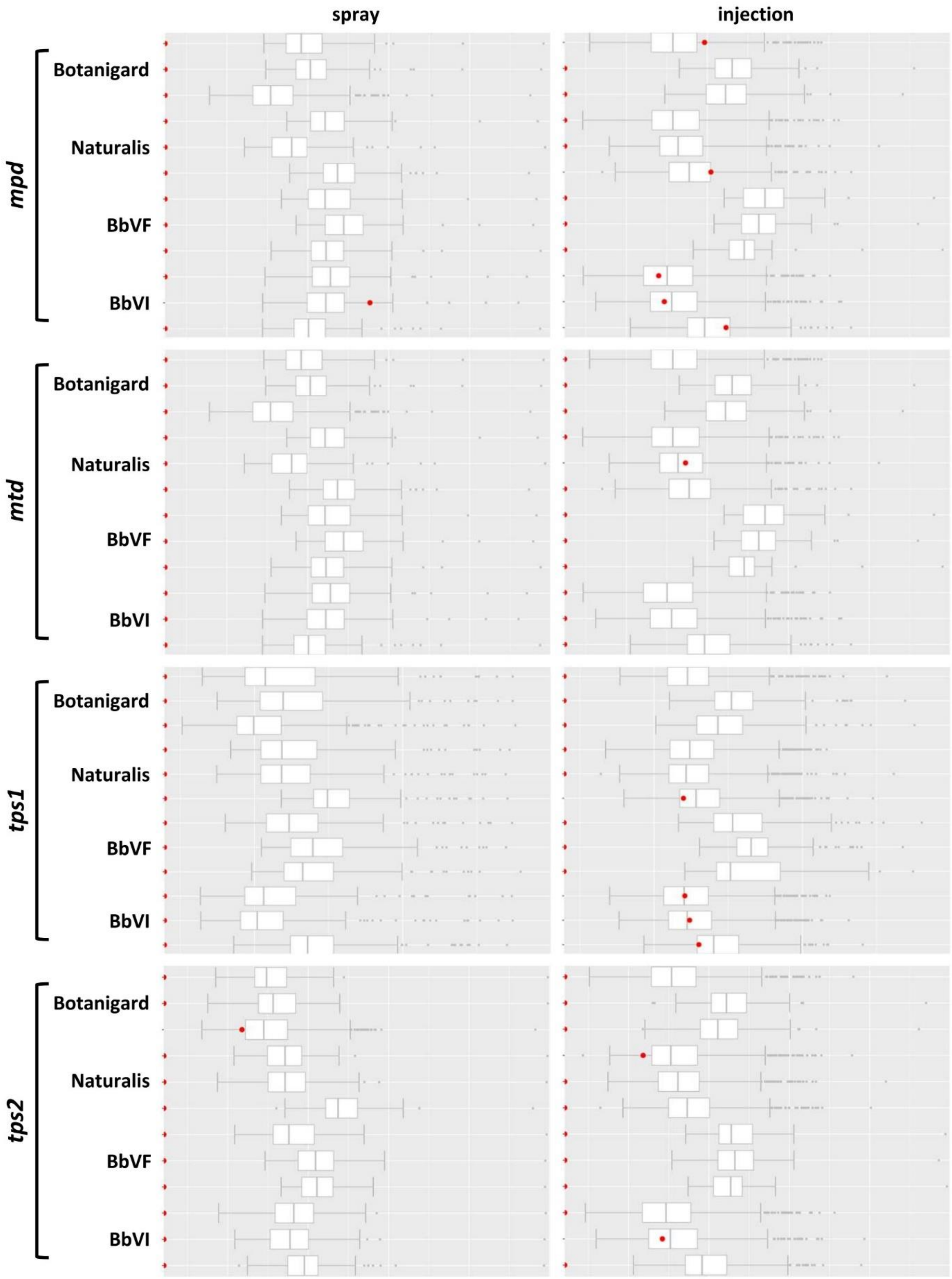

Figure S24: Boxplots demonstrating the expression profiles of fungal metabolism genes *mpd*, *mtd*, *tps1* and *tps2* following infection of *T. molitor* at 48 dpi.
